# A phylogenetic framework of the legume genus *Aeschynomene* for comparative genetic analysis of the Nod-dependent and Nod-independent symbioses

**DOI:** 10.1186/s12870-018-1567-z

**Published:** 2018-12-05

**Authors:** Laurent Brottier, Clémence Chaintreuil, Paul Simion, Céline Scornavacca, Ronan Rivallan, Pierre Mournet, Lionel Moulin, Gwilym P. Lewis, Joël Fardoux, Spencer C. Brown, Mario Gomez-Pacheco, Mickaël Bourges, Catherine Hervouet, Mathieu Gueye, Robin Duponnois, Heriniaina Ramanankierana, Herizo Randriambanona, Hervé Vandrot, Maria Zabaleta, Maitrayee DasGupta, Angélique D’Hont, Eric Giraud, Jean-François Arrighi

**Affiliations:** 1 IRD, Laboratoire des Symbioses Tropicales et Méditerranéennes, UMR LSTM, Campus International de Baillarguet, 34398 Montpellier, France; 2 Institut des Sciences de l’Evolution (ISE-M), Université de Montpellier, CNRS, IRD, EPHE, 34095 Cedex 5 Montpellier, France; 3 CIRAD (Centre de Coopération Internationale en Recherche Agronomique pour le Développement), UMR AGAP, F-34398 Montpellier, France; 4 AGAP,Univ Montpellier, CIRAD, INRA, Montpellier SupAgro, 34060 Montpellier, France; 5 IRD, Interactions Plantes Microorganismes Environnement, UMR IPME, 34394 Montpellier, France; 6 Comparative Plant and Fungal Biology Department, Royal Botanic Gardens, Kew, Richmond, Surrey TW9 3AB UK; 7 Institute of Integrative Biology of the Cell (I2BC), CEA, CNRS, Univ. Paris-Sud, Université Paris-Saclay, 91198 Gif-sur-Yvette, France; 8 Laboratoire de Botanique, Institut Fondamental d’Afrique Noire, Ch. A. Diop, BP 206 Dakar, Sénégal; 9 Laboratoire de Microbiologie de l’Environnement/Centre National de Recherche sur l’Environnement, 101 Antananarivo, Madagascar; 10 IAC, Laboratoire de Botanique et d’Ecologie Végétale Appliquée, UMR AMAP, 98825 Pouembout, Nouvelle-Calédonie France; 11 Department of Biochemistry and Microbial Genomics, IIBCE, 11600 Montevideo, Uruguay; 12 Department of Biochemistry, University of Calcutta, Kolkata, 700019 India

**Keywords:** *Aeschynomene*, Genetics, Legumes, Nodulation, Phylogenetics, Polyploidy, Symbiosis

## Abstract

**Background:**

Among semi-aquatic species of the legume genus *Aeschynomene*, some have the property of being nodulated by photosynthetic *Bradyrhizobium* lacking the *nodABC* genes necessary for the synthesis of Nod factors. Knowledge of the specificities underlying this Nod-independent symbiosis has been gained from the model legume *Aeschynomene evenia* but our understanding remains limited due to the lack of comparative genetics with related taxa using a Nod factor-dependent process. To fill this gap, we combined different approaches to perform a thorough comparative analysis in the genus *Aeschynomene*.

**Results:**

This study significantly broadened previous taxon sampling, including in allied genera, in order to construct a comprehensive phylogeny. In the phylogenetic tree, five main lineages were delineated, including a novel lineage, the Nod-independent clade and another one containing a polytomy that comprised several *Aeschynomene* groups and all the allied genera. This phylogeny was matched with data on chromosome number, genome size and low-copy nuclear gene sequences to reveal the diploid species and a polytomy containing mostly polyploid taxa. For these taxa, a single allopolyploid origin was inferred and the putative parental lineages were identified. Finally, nodulation tests with different *Bradyrhizobium* strains revealed new nodulation behaviours and the diploid species outside of the Nod-independent clade were compared for their experimental tractability and genetic diversity.

**Conclusions:**

The extended knowledge of the genetics and biology of the different lineages sheds new light of the evolutionary history of the genus *Aeschynomene* and they provide a solid framework to exploit efficiently the diversity encountered in *Aeschynomene* legumes. Notably, our backbone tree contains all the species that are diploid and it clarifies the genetic relationships between the Nod-independent clade and the Nod-dependent lineages. This study enabled the identification of *A. americana* and *A. patula* as the most suitable species to undertake a comparative genetic study of the Nod-independent and Nod-dependent symbioses.

**Electronic supplementary material:**

The online version of this article (10.1186/s12870-018-1567-z) contains supplementary material, which is available to authorized users.

## Background

In the field of nitrogen-fixing symbiosis, scientists have a long-standing interest in the tropical papilionoid legume genus *Aeschynomene* since the discovery of the ability of the species *A. afraspera* to develop abundant stem nodules [1]. This nodulation behavior is uncommon in legumes, being shared by very few hydrophytic species of the genera *Discolobium*, *Neptunia* and *Sesbania*, but it is exceptionally widespread among the semi-aquatic *Aeschynomene* species [2–4]. These stem-nodulating *Aeschynomene* species are able to interact with *Bradyrhizobium* strains that display the unusual property to be photosynthetic [5, 6]. However, most outstanding is the evidence that some of these photosynthetic *Bradyrhizobium* strains lack both the *nodABC* genes required for the synthesis of the key “Nod factors” symbiotic signal molecules and a type III secretion system (T3SS) that is known in other rhizobia to activate or modulate nodulation [7–9]. These traits revealed the existence of an alternative symbiotic process between rhizobia and legumes that is independent of the Nod factors.

As in the legume genus *Arachis* (peanut), *Aeschynomene* uses an intercellular symbiotic infection process instead of infection thread formation that can be found in other legume groups [10]. This lead to the suggestion that the Nod-independent process might correspond to the ancestral state of the rhizobial symbiosis although it cannot be excluded it corresponds to an alternative symbiotic interaction compared to the one described in other legumes [11–13]. It is noteworthy that all the Nod-independent species form a monophyletic clade within the *Aeschynomene* phylogeny and jointly they also display striking differences in the bacteroid differentiation process compared to other *Aeschynomene* species [4, 14]. To decipher the molecular mechanisms of this distinct symbiosis, the Nod-independent *A. evenia* has been taken as a new model legume, because its genetic and developmental characteristics (diploid with a reasonable genome size -2n = 20, 415 Mb/1C-, short perennial and autogamous, can be hybridized and transformed) make this species tractable for molecular genetics [15–17]. Functional analyses revealed that some symbiotic determinants identified in other legumes (*SYMRK*, *CCaMK*, *HK1* and *DNF1*) are recruited, but several key genes involved in bacterial recognition (e.g. *LYK3*), symbiotic infection (e.g. *EPR3* and *RPG*), and nodule functioning (e.g. *DNF2* and *FEN1*) were found not to be expressed in *A. evenia* roots and nodules, based on RNAseq data [14, 18–20]. This suggested that the Nod-independent symbiosis is distinct from the Nod-dependent one.

Forward genetics are now expected to allow the identification of the specific molecular determinants of the Nod-independent process in *A. evenia* [15, 19]. In addition, comparing *A. evenia* with closely related Nod-dependent *Aeschynomene* species will promote our understanding how the Nod-independent symbiosis evolved in *Aeschynomene*. The genus *Aeschynomene* (restricted now to the section *Aeschynomene* as discussed in [4]) is traditionally composed of three infrageneric taxa, subgenus *Aeschynomene* (which includes all the hydrophytic species) and subgenera *Bakerophyton* and *Rueppellia* [21, 22]. The genus has also been shown to be paraphyletic, a number of related genera being nested within, but altogether they form a distinct clade in the tribe Dalbergieae [4, 23–26]. Within this broad clade, two groups of semi-aquatic *Aeschynomene* have been well-studied from a genetic and genomic standpoint: the *A. evenia* group, which contains all the Nod-independent species (most of them being 2x), and the *A. afraspera* group (all species being Nod-dependent) that appears to have a 4x origin [27–29]. For comparative analyses, the use of Nod-dependent species with a diploid structure would be more appropriate, but such *Aeschynomene* species are poorly documented.

To overcome these limitations, we aimed to produce a species-comprehensive phylogenetic tree supplemented with genetic and nodulation data. For this, we made use of an extensive taxon sampling in both the genus *Aeschynomene* and in closely related genera to capture the full species diversity of the genus and to clarify phylogenetic relationships between taxa. For most species, we also documented chromosome number, genome size and molecular data for low-copy nuclear genes, thus allowing the identification of diploid species as well as untangling the genome structure of polyploid taxa. In addition, these species were characterized for their ability to nodulate with various *Bradyrhizobium* strains containing or lacking *nod* genes and finally, the diploid species were submitted to a comparative analysis of their properties. In the light of the data obtained in this study, we propose two complementary *Aeschynomene* species to set a comparative genetic system with the *A. evenia* model.

## Results

### A comprehensive phylogeny of the genus Aeschynomene and allied genera

To obtain an in-depth view of the phylogenetic relationships within the genus *Aeschynomene* subgenus *Aeschynomene*, which contains the hydrophytic species, we significantly increased previous sampling levels by the addition of new germplasm accessions and, if these were not available, we used herbarium specimens. This strategy allowed checking the species identity and obtaining complementary data on the same plant material. DNA was isolated for 40 out of the 41 species (compared to the 27 species used in [4]) included in this group in taxonomic and genetic studies (Additional file 1: Table S1) [4, 21, 27–29]. In addition, to determine the phylogenetic relationship of this subgenus with *Aeschynomene* subgenera *Bakerophyton* and *Rueppellia*, unclassified *Aeschynomene* species, as well as with the allied genera *Bryaspis*, *Cyclocarpa*, *Geissaspis*, *Humularia*, *Kotschya*, *Smithia* and *Soemmeringia*, we also sampled all these 10 taxa (compared to the 5 taxa present in [4]) [23, 30]. This added 21 species to our total samples (Additional file 1: Table S1). The dalbergioid species *Pictetia angustifolia* was used as outgroup [4, 26].

**Fig. 1:**
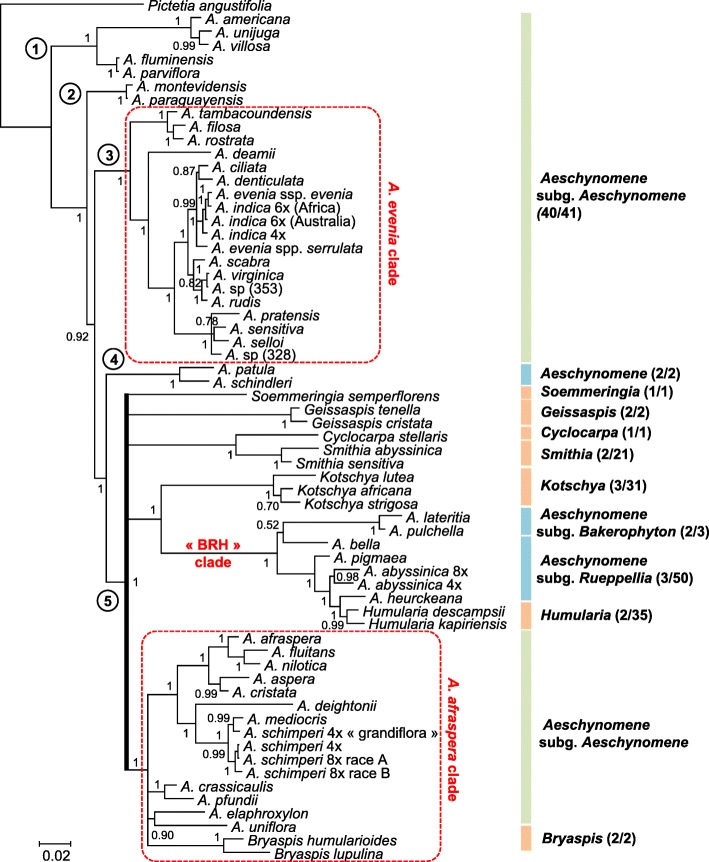
Phylogeny of the genus *Aeschynomene* and allied genera. The Bayesian phylogenetic reconstruction was obtained using the concatenated *ITS* (Internal Transcribed Spacer) + *matK* sequences. Numbers at branches indicate posterior probability above 0.5. The five main lineages are identified with a circled number and the two previously studied *Aeschynomene* groups are framed in a red box bordered with a dashed line. On the right are listed *Aeschynomene* subgenus *Aeschynomene* (in green), other *Aeschynomene* subgenera or species groups (in blue) and related genera (in orange) with numbers of sampled species/total species indicated into parenthesis

Phylogenetic reconstruction of all the taxa sampled was undertaken using Bayesian analysis of the chloroplast *matK* gene and the nuclear ribosomal *ITS* region that were processed separately (Additional file 2: Table S2, Additional file 3: Table S3). The *matK* and *ITS* sequences produced Bayesian trees that distinguished almost all the different *Aeschynomene* groups and related genera (Additional file 4: Figure S1; Additional file 5: Figure S2). The two phylogenetic trees have a very similar topology although some branches can be lowly supported in one of them. Incongruences were also observed for *A. deamii* and the genus *Bryaspis*, but the conflicting placements are lowly supported and so, they were interpreted as lack of resolution rather than hard incongruence. To improve the phylogenic resolution among the major lineages, the *matK* gene and the *ITS* sequence datasets were combined into a single phylogenetic analysis where only well-supported nodes were considered (posterior probability (PP) ≥ 0.5) (Fig. 1). Our analysis recovered a grade of five main lineages with a branching order that received robust support (PP ≥ 0.92): (1) a basally branching lineage including *A. americana*, (2) an *A. montevidensis* lineage, (3) an *A. evenia* lineage corresponding to the Nod-independent clade [15, 27], (4) a new-identified lineage containing *A. patula* and (5) a lineage represented by an unresolved polytomy gathering the *A. afraspera* clade [19] and all the remaining taxa.

Our work also provided in the main good species-level resolution and it showed that *Aeschynomene* subgenus *Aeschynomene* (as currently circumscribed) is polyphyletic, being interspersed on the phylogenetic tree with the lineage containing *A. patula*, the two other subgenera of *Aeschynomene* and a number of other genera related to *Aeschynomene* (Fig. 1) [4, 24, 26, 31]. The combined analysis also grouped the genus *Bryaspis* with the species related to *A. afraspera* in a highly supported clade but it remained inconclusive regarding its exact positioning as previously observed in a *trnL*-based phylogeny (Fig. 1) [4]. Most noticeably, several intergeneric relationships are consistently revealed, notably between *Cyclocarpa* and *Smithia* as well as in the clade containing *Aeschynomene* subgenera *Bakerophyton* and *Rueppellia* together with the genus *Humularia* (referred to as the BRH clade herein after) (Fig. 1). This clade supports previous observations of a morphological continuum between *Aeschynomene* subgenus *Rueppellia* and the genus *Humularia* and brings into question their taxonomic separation [22].

### Ploidy level of the species and origin of the polyploid lineages

**Fig. 2:**
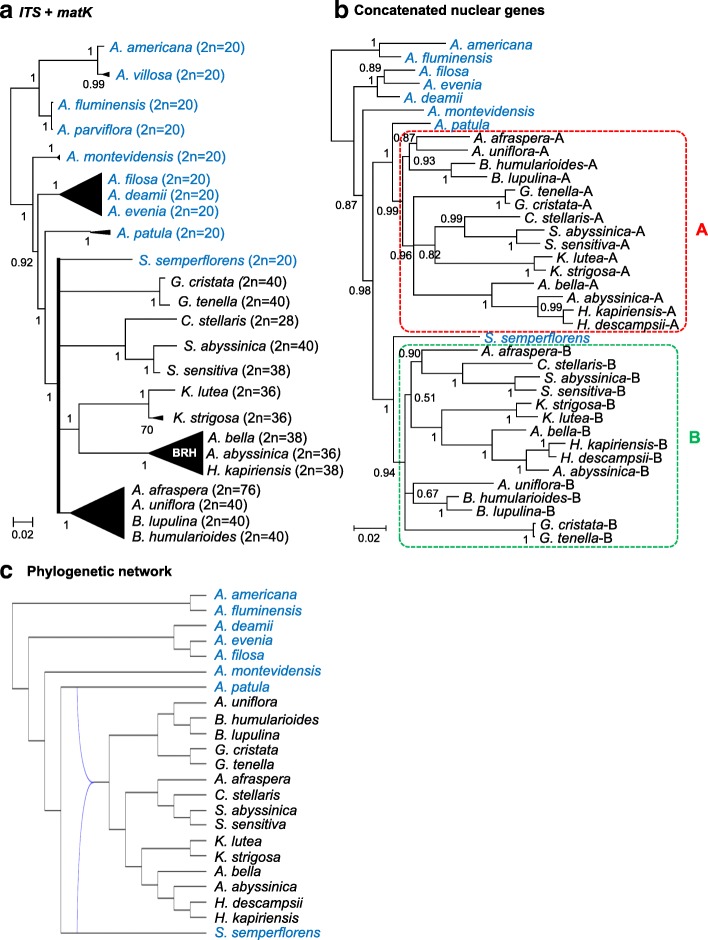
Genomic characteristics and phylogenetic relationships. **a** Simplified Bayesian *ITS* + *matK* phylogeny with representative species of different lineages and groups. The *A. evenia*, *A. afraspera* and BRH (*Bakerophyton-Rueppelia-Humularia*) clades are represented by black triangles and the polytomy is depicted in bold. Chromosome numbers are indicated in brackets. **b** Phylogenetic relationships based on the combination of 4 concatenated nuclear low-copy genes (*CYP1*, *eif1a*, *SuSy* and *TIP1;1* genes detailed in Additional file 8: Figure S5). Diploid species (2n = 20) are in blue, polyploid species (2n ≥ 28) in black. The A and B subgenomes of the polyploid taxa are delineated by red and green boxes in dashed lines, respectively. Nodes with a posterior probability inferior to 0.5 were collapsed into polytomies. Posterior probability above 0.5 are indicated at every node. **c** The one-allopolyploidation hypothesis (N1-best) obtained with the phylogenetic network analysis based on the T2 tree with reticulations in blue (detailed in Additional file 10: Figure S7)

The revised *Aeschynomene* phylogeny was used as a backbone tree to investigate the genetic status of the different species and the evolution of ploidy levels. Previous studies had demonstrated that the *A. evenia* clade is mostly diploid (2n = 2x = 20) even if some species such as *A. indica* (2n = 4x = 40, 2n = 6x = 60) appear to be of recent allopolyploid origin [27, 29]. Conversely, all the species of the *A. afraspera* group were found to be polyploid (2n = 4x = 28,38,40, 2n = 8x = 56,76) and to have a common AB genome structure but the origin of the polyploidy event remained undetermined [28]. To assess the ploidy levels in *Aeschynomene* species and related genera, chromosome numbers and nuclear DNA content were determined (appended to labels in Fig. 2 a, Additional file 1: Table S1, Additional file 6: Figure S3 and Additional file 7: Figure S4). We evidenced the lineages containing *A. americana*, *A. montevidensis*, *A. evenia* and *A. patula*, as well as *Soemmeringia semperflorens*, to be diploid with 2n = 20, with the smallest 2x genome for *A. patula* (0.58 pg/2C) and the largest 2x genome for *A. deamii* (1.93 pg/2C). With the exception of *S. semperflorens*, all the groups that are part of the polytomy were characterized by higher chromosome numbers. These chromosome numbers equate to approximately twice the one present in diploid species (except for 2 = 28), suggesting that the corresponding groups are most probably polyploid. Putatively polyploid species with chromosome numbers departing from 2n = 40 are likely to be of disploid origin as already described in the *A. afraspera* clade [28]. Here again, important genome size variations ranging from 0.71 pg/2C for the *Geissaspis* species to 4.82 pg/2C for the 4x *A. schimperi* highlight the genomic differentiation of the various taxa (Fig. 2 a, Additional file 1: Table S1).

To firmly link chromosome numbers to ploidy levels and to clarify genetic relationships between the different lineages, we cloned and sequenced four nuclear-encoded low-copy genes in selected species: *CYP1* (Cyclophilin 1), *eiF1α* (eukaryotic translation initiation factor α), *SuSy* (Sucrose Synthase) and *TIP1;1* (tonoplast intrinsic protein 1;1) (Additional file 2: Table S2). For all diploid species, only one gene sequence was obtained, while for all the polyploid species, in almost all cases, a pair of putative homeologues was isolated, thus confirming their genetic status inferred from the karyotypic data (Additional file 3: Table S3). In general, the duplicated copies were highly divergent and nested in two different major clades in the resulting Bayesian phylogenic trees generated for each gene (Additional file 8: Figure S5). One clade contained all the A copies (except for one anomalous sequence for *B. lupulina* in the *eiF1α* tree) and the other clade gathered all the B copies previously identified in *A. afraspera* [28]. These two clades A and B do not always receive high support, however it is notable that the A copies formed a monophyletic group with, or sister to, the *A. patula* sequence and similarly the B copies with, or sister to, the *S. semperflorens* sequence, in all gene trees (Additional file 8: Figure S5). In an attempt to improve phylogenetic resolution, the four gene data sets were concatenated. This combination resulted in a highly supported Bayesian tree that places the A copy clade as the sister to the diploid *A. patula* (PP =1), and the B copy clade as sister to the diploid *S. semperflorens* (PP =1) (Fig. 2 b). As a result, these phylogenetic analyses combined to karyotypic data show that all the five main lineages contain diploid species. They also reveal that all the polyploid groups share the same AB genome structure, with the diploid *A. patula* and *S. semperflorens* species being the modern representatives of the ancestral donors of the A and B genomes.

In addition, an ancestral state reconstruction analysis performed on the *ITS* + *matK* phylogeny indicates that diploidy is the ancestral condition in the whole revised group and that tetraploidy most likely evolved once in the polytomy (Additional file 9: Figure S6). To provide support on a probable single origin of the allopolyploidy event, separate and concatenated nuclear gene trees were further used for a phylogenetic network analysis. In this analysis, the two non-allopolyploidisation hypotheses (T1 and T2) were found to be more costly (scores of 207 and 196) than the two hypotheses allowing for hybridization (N1-best and N2-best with scores of 172 and 169, respectively) (Additional file 10: Figure S7a-d). The one-allopolyploidisation hypothesis (N1-best) strongly indicates that a hybridization between *A. patula* and *S. semperflorens* gave rise to the polyploid lineages as inferred above (Fig. 2c, Additional file 10: Figure S7c). Although the two-allopolyploidisation hypothesis (N2-best) yielded the absolute best score, the score improvement was very low (169 vs 172) and the resulting network included the hybridization inferred with the one-allopolyploidisation hypothesis making this latter hypothesis most probably the correct one (Additional file 10: Figure S7d).

### Nodulation properties of the different Aeschynomene lineages

**Fig. 3:**
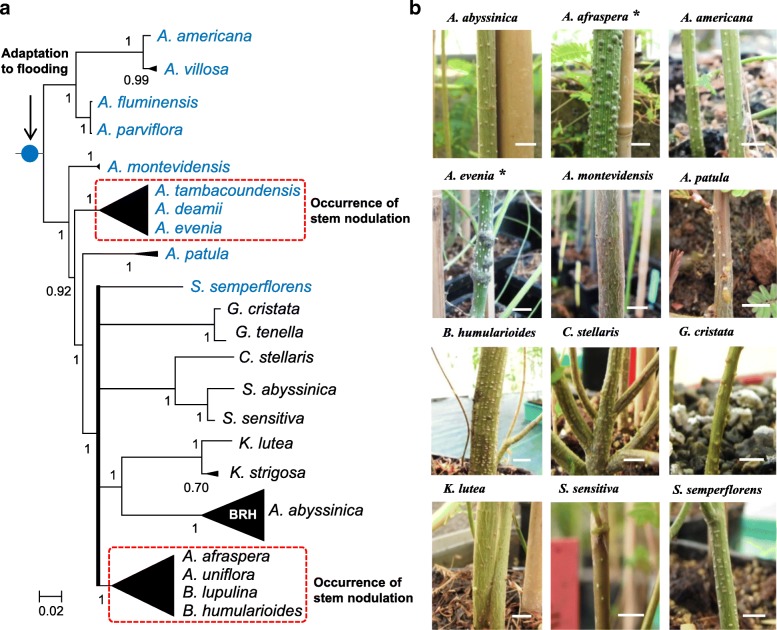
Occurrence of adventitious root primordia and of stem nodulation. **a** Simplified Bayesian *ITS* + *matK* phylogeny of the whole group with the *A. evenia*, *A. afraspera* and BRH (*Bakerophyton-Rueppelia-Humularia*) clades represented by black triangles. The polytomy is depicted in bold. The shared presence of adventitious root primordia is depicted on the stem by a blue circle. Dashed red boxes indicate groups comprising aerial stem-nodulating species. Asterisks refer to illustrated species in (**b**) for aerial stem-nodulation. **b** Stems of representatives for the different lineages and groups. Small spots on the stem correspond to dormant adventitious root primordia and stem nodules are visible on the species marked by an asterisk. Bars: 1 cm

Species of *Aeschynomene* subgenus *Aeschynomene* are known to be predominantly amphibious and more than 15 of such hydrophytic species (found in the *A. evenia* and *A. afraspera* clades, as well as *A. fluminensis*) have been described as having the ability to develop stem nodules [3, 21, 28, 32]. In *A. fluminensis*, these nodules are observed only in submerged conditions (as also seen in the legume *Discolobium pulchellum*), while they occur on aerial stems within the *A. evenia* and *A. afraspera* clades (Fig. 3 a) [4, 33–35]. Phenotypic analysis of representatives of the different lineages under study revealed that they all display adventitious root primordia along the stem (Fig. 3 a,b). Adventitious roots are considered to be an adaptation to temporary flooding and they also correspond to nodulation sites in stem-nodulating *Aeschynomene* species (Fig. 3 b) [35]. Given that the *A. evenia* and *A. afraspera* clades are now demonstrated to have different genomic backgrounds provides a genetic argument for independent developments of stem nodulation by photosynthetic bradyrhizobia. Reconstruction of ancestral characters based on the *ITS* + *matK* phylogeny confirmed that the whole group was ancestrally of wet ecology and endowed with adventitious root primordia but that the stem nodulation ability evolved several times as previously inferred (Additional file 11: Figure S8; Additional file 12: Figure S9; Additional file 13: Figure S10) [4, 28].

**Fig. 4:**
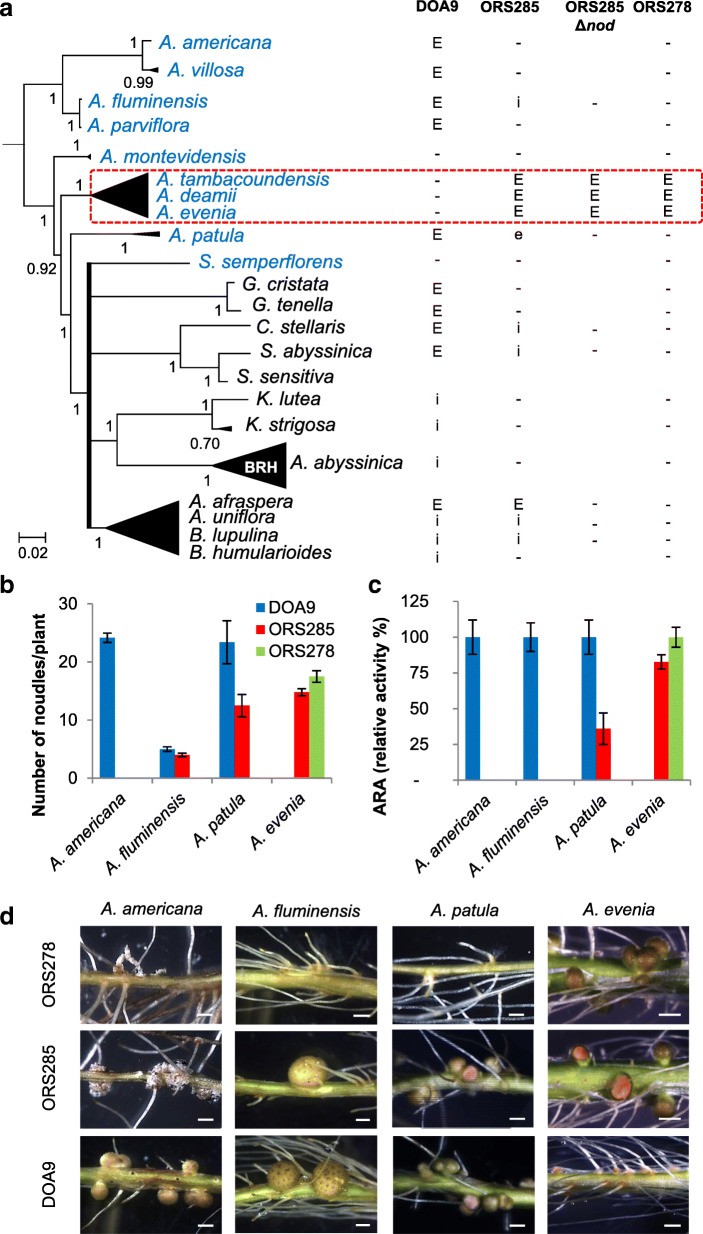
Comparison of the root nodulation properties. **a** Species of different lineages and groups that were tested for nodulation are listed in the simplified Bayesian phylogeny on the left. Root nodulation tests were performed using the DOA9, ORS285, ORS285∆*nod* and ORS278 strains. E, effective nodulation; e, partially effective nodulation; i, ineffective nodulation, −, no nodulation; blank, not tested. **b** Number of nodules per plant, **c** relative acetylene-reducing activity (ARA) and **d** aspect of the inoculated roots developing nodules or not (some nodules were cut to observe the leghemoglobin color inside) after inoculation with *Bradyrhizobium* DOA9, ORS285 and ORS278 on *A. americana*, *A. patula*, *A. afraspera* and *A. evenia*. Error bars in (**b**) and (**c**) represent s.d. (*n* = 6). Scale bar in (**d**): 1 mm

To investigate whether the newly studied species could be nodulated by photosynthetic bradyrhizobia, we extended the results obtained by Chaintreuil et al. [4] by testing the nodulation abilities of 22 species available (listed in Fig. 4 a) for which sufficient seeds were available. Three different strains of *Bradyrhizobium* equating to the three cross-inoculation (CI) groups defined by Alazard [2] were used: DOA9 (non-photosynthetic *Bradyrhizobium* of CI-group I), ORS285 (photosynthetic *Bradyrhizobium* with *nod* genes of CI-group II) and ORS278 (photosynthetic *Bradyrhizobium* lacking *nod* genes of CI-group III). These strains were used to inoculate the 22 species and their ability to nodulate them was analyzed at 21 dpi. For this, we recorded nodule formation and compared nitrogen fixation efficiency by an acetylene reduction assay (ARA) and observation of plant vigor. Nodulation was observed on all species tested except for *S. sensitiva* that had problem of root development, for *A. montevidensis* and *S. semperflorens*. For these three species, either the culture conditions or the *Bradyrhizobium* strains used were not appropriate (Fig. 4 a).

The non-photosynthetic strain DOA9 displayed a wide host spectrum but was unable to nodulate the Nod-independent species, *A. deamii*, *A. evenia* and *A. tambacoundensis*. The photosynthetic strain ORS285 efficiently nodulated *A. afraspera* and the Nod-independent *Aeschynomene* species (Fig. 4 a), as previously reported [4]. Interestingly, the ORS285 strain was also able to induce nitrogen-fixing nodules in *A. patula* and ineffective nodules were observed on *A. fluminensis* and the genera *Bryaspis*, *Cyclocarpa* and *Smithia* (Fig. 4 a). To examine if in these species the nodulation process relies on a Nod-dependent or Nod-independent symbiotic process, we took advantage of the availability of a ∆*nod* mutant of the strain ORS285. None of them were found to be nodulated by ORS285∆*nod*, suggesting that the nodule formation depended on a Nod signaling in these species (Fig. 4 a). As a matter of fact, the ORS285∆*nod* mutated strain was found to be able to nodulate only species of the *A. evenia* clade similarly as to the photosynthetic strain ORS278 naturally lacking *nod*-genes (Fig. 4 a). Analysis of the evolution of these nodulation abilities by performing an ancestral state reconstruction on the revisited phylogeny indicated several emergences of the ability to interact with photosynthetic bradyrhizobia and a unique emergence of the ability to be nodulated by the *nod* gene-lacking strain as observed earlier (Additional file 14: Figure S11; Additional file 15: Figure S12) [4]. Finally, from these nodulation tests, different nodulation patterns emerged for the diploid *Aeschynomene* species (as detailed in Fig. 4 b-d) with the DOA9 and ORS278 strains being specific to the Nod-dependent and Nod-independent groups respectively and ORS285 showing a gradation of compatibility between both.

### Diversity of the diploid species outside the nod-independent clade

**Fig. 5:**
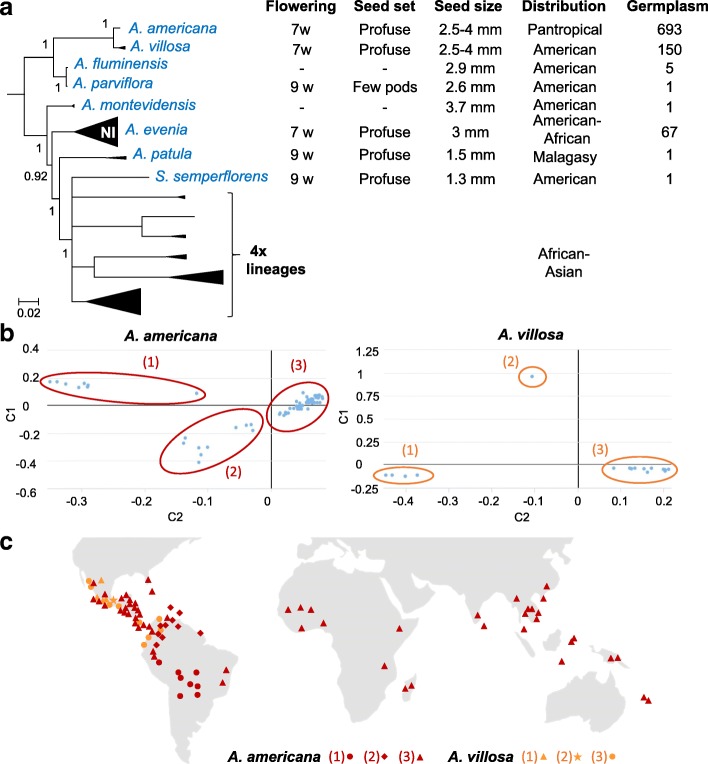
Characteristics of diploid species. **a** Development and germplasm data for species that are listed in the simplified phylogeny on the left. *A. evenia* from the Nod-independent clade (NI) is also included for comparison. Germplasm numbers correspond to the sum of accessions available at CIAT, USDA, Kew Gardens, AusPGRIS, IRRI and at LSTM. **b** Multi-dimensional scaling (MSD) plots of the genetic diversity among *A. americana* (left) and *A. villosa* (right) accessions according to coordinates 1 and 2 (C1, C2). Identified groups are delimited by circles and labeled with numbers. **c** Geographical distribution of the of *the A. americana* and *A. villosa* accessions. Taxon colours and group numbers are the same as in (**b**). Details of the accessions are provided in Additional file 16: Table S4. Word map from https://pixabay.com

To further characterize the diploid species that fall outside of the Nod-independent clade, in which *A. evenia* relies, they were analyzed for their developmental properties and genetic diversity (Fig. 5 a). All species are described as annual or short perennial [21, 30, 31]. While *A. americana*, *A. villosa*, *A. fluminensis*, *A. parviflora* and *A. montevidensis* are robust and erect, reaching up to 2 m high when mature similarly as to *A. evenia*, *A. patula* and *S. semperflorens* are creeping or decumbent herbs. These differences in plant habit is reflected by the important variation in seed size between these two groups (Fig. 5 a). This has an impact on plant manipulation since for *A. patula* and *S. semperflorens* seed scarification needs to be adapted (25 min with concentrated sulfuric acid instead of 40 min for the other species) and in vitro plant growth takes slightly more time to get a root system sufficiently developed for inoculation with *Bradyrhizobium* strains (10 days-post-germination instead of the 5–7 dpi for other species) [15]. Consistent flowering and seed production was observed for *A. americana*, *A. villosa*, *A. patula* and *S. semperflorens* when grown under full ambient light in the tropical greenhouse in short days conditions as previously described for *A. evenia*, making it possible to develop inbred lines by successive selfing (Fig. 5 a) [15]. For *A. fluminensis*, *A. parviflora* and *A. montevidensis*, flowering was sparse or not observed, indicating that favorable conditions for controlled seed set were not met (Fig. 5 a).

Five species (*A. villosa*, *A. fluminensis*, *A. parviflora*, *A. montevidensis* and *S*. *semperflorens*) are strictly American while *A. americana* is a pantropical species and *A. patula* is endemic to Madagascar [21, 31, 32]. Several species have a narrow geographic distribution or seem to be infrequent, explaining the very limited accession availability in seedbanks (Fig. 5 a) [21, 31, 32]. This is in sharp contrast with both *A. americana* and *A. villosa* that are well-collected, being widely found as weedy plants and sometimes used as component of pasture for cattle (Fig. 5 a) [36]. To assess the genetic diversity of these two species, a germplasm collection containing 79 accessions for *A. americana* and 16 accessions for *A. villosa*, and spanning their known distribution was used (Additional file 16: Table S4). A Genotyping-By-Sequencing (GBS) approach resulted in 6370 and 1488 high quality polymorphic SNP markers for *A. americana* and *A. villosa* accessions, respectively. These two SNP datasets subsequently served for a clustering analysis based on the multidimensional-scaling (MSD) method. The MSD analysis distinguished three major groups of accessions for both *A. americana* and *A. villosa* along coordinate axes 1 and 2 (Fig. 5 b). When mapping the accessions globally, the three groups identified for *A. villosa* were observed conjointly in Mexico and only the group (3) extended to the northern part of South America (Fig. 5c, Additional file 16: Table S4). Contrarily, a clear geographical division was observed for *A. americana* with the group (1) occupying the central part of South America, group (2) being found in the upper part of South America while group (3) was present in distinct regions from Mexico to Brazil and in all the Paleotropics (Fig. 5c, Additional file 16: Table S4). *A. americana* is hypothesized to be native in America and naturalized elsewhere [36]. The observed distributions in combination with the fact that in the MSD analysis accessions are tightly clustered in group (3) compared to groups (1) and (2) support this idea and indicate that its group (3) recently spread worldwide.

## Discussion

### A well-documented phylogenetic framework for the legume genus Aeschynomene

We produced a new and comprehensive phylogeny of the genus *Aeschynomene* and its closely related genera complemented by gene data sets, genome sizes, karyotypes and nodulation assays. For plant genera, they are few for which documentation of taxonomic diversity is that extensive and supported by a well-resolved, robustly supported phylogeny so as to reveal the evolutionary history of these groups [37]. Here, the whole group, which includes the genus *Aeschynomene* with its 3 subgenera and its 7 allied genera, is evidenced to comprise five main lineages, including the Nod-independent clade, with diploid species that could be found in all these lineages. The multigene data analysis provided robust evidence that two of them, represented by the two diploid species *A. patula* and *S. semperflorens*, are involved in an ancient allotetraploidization process that gave rise to the different polyploid lineages clustering in a polytomy. Separate allopolyploidization events from the same diploid parents or a single allopolyploid origin are plausible explanations for the formation of these lineages. However, the consistent resolution of the phylogenetic tree obtained with the combined gene data, where *A. patula* and *S. semperflorens* are sisters to the A and B subgenomic sequences, favours the hypothesis of a single allopolyploid origin, as also argued for other ancient plant allopolyploid events in *Asimitellaria* (Saxifragaceae) and *Leucaena* (Leguminosae) [37, 38]. The phylogenetic network analysis also supports the one-allopolyploidisation hypothesis. However, additional nuclear genes will be needed to conclusively confirm that no additional hybridization event occurred. Although not the focus of the present study, it is worth noting that most diploid species are found in the Neotropics, the two modern representatives of the A and B genome donors that gave rise to the 4x lineages are located on different continents (*S. semperflorens* in South America and *A. patula* in Madagascar) and that all the 4x lineages are located in the Paleotropics [30]. This raises questions about the evolution of the whole group and the origin of the 4x lineages. In addition, the presence of a polytomy suggests that this allopolyploid event preceded a rapid and major diversification of 4x groups that have been ascribed to different *Aeschynomene* subgenera or totally distinct genera that altogether represent more than 80% of the total species of the whole group [26, 39]. Diversification by allopolyploidy occurred repeatedly in the genus *Aeschynomene* since several neopolyploid species were evidenced in both the *A. evenia* clade and the *A. afraspera* clade as exemplified by *A. indica* (4x, 6x) and *A. afraspera* (8x) [27, 28]. Dense sampling for several *Aeschynomene* taxa or clades also allowed delimiting more precisely species boundaries (for morphologically similar taxa but which are genetically differentiated or correspond to different cytotypes) and evidencing intraspecific genetic diversity that is often geographically-based as showed for the pantropical species *A. americana* (this study), *A. evenia*, *A. indica* and *A. sensitiva* [29]. All these *Aeschynomene* share the presence of adventitious root primordia on the stem that correspond to the infection sites for nodulation. The ever-presence of adventitious root primordia in all taxa of the whole group and an ancestral state reconstruction substantiate the two-step model proposed earlier for the evolution of stem nodulation in *Aeschynomene* with a common genetic predisposition at the base of the whole group to produce adventitious root primordia on the stem, as an adaptation to flooding, and subsequent mutations occurring independently in various clades to enable stem nodulation [4]. The ability to interact with photosynthetic bradyrhizobia that are present in aquatic environments also appear to have evolved at least 3 times [4 and this work, Fig. 4]. This photosynthetic activity is important for the bacterial symbiotic lifestyle as it provides energy usable for infection and subsequently for nitrogenase activity inside the stem nodules [5]. To date, natural occurrence of nodulation by photosynthetic bradyrhizobia has been reported only for the *A. evenia* and *A. afraspera* clades, and for *A. fluminensis* [6, 34, 40]. Nevertheless, we could not test the photosynthetic strains isolated from *A. fluminensis* nodules and the nature of the strains present in those of the newly studied species *A. patula* has not been investigated yet. They would allow the comparison of their nodulation efficiency with the reference photosynthetic *Bradyrhizobium* ORS278 and ORS285 strains. In addition, we can ask if the semi-aquatic lifestyle and/or nodulation with photosynthetic bradyrhizobia may have facilitated the emergence of the Nod-independent symbiosis in the *A. evenia* clade.

### Aeschynomene species for a comparative analysis of nodulation with *A. evenia*

To uncover whether the absence of detection for several key symbiotic genes in the root and nodule transcriptomic data of *A. evenia* are due to gene loss or inactivation, and to identify the specific symbiotic determinants of the Nod-independent symbiosis, a genome sequencing combined to a mutagenesis approach is presently being undertaken for *A. evenia* in our laboratory. A comparative analysis with Nod-dependent *Aeschynomene* species is expected to consolidate this genomic and genetic analysis performed in *A. evenia* by contributing to elucidate the genetic changes that enabled the emergence of the Nod-independent process. Phylogenomics and comparative transcriptomics, coupled with functional analysis, are undergoing increased development in the study of symbiosis to unravel gene loss linked to the lack of developing a symbiosis but also to identify new symbiosis genes (for arbuscular mycorrhizal symbiosis [41, 42]; for the nodulating symbiosis [43, 44]). Comparative work on symbiotic plants is often hindered, however, either by the absence of closely related species which display gain or loss of symbiotic function or, when these are present, by the lack of well-understood genetic framework, as outlined in [10, 43, 45, 46]. In fact, such situations are few, but in the case of the nodulating *Parasponia*/non-nodulating *Trema* system, a fine comparative analysis was very powerful to evidence a parallel loss of the key symbiotic genes *NFP2*, *NIN* and *RGP,* in the non-nodulating species, challenging the long-standing assumption that *Parasponia* specifically acquired the potential to nodulate [45–47]. In this respect, the uncovering of the genetic evolution of the genus *Aeschynomene* and related genera along with the identification of diploid species outside of the Nod-independent clade, provided a robust phylogenetic framework that can now be exploited to guide the choice of Nod-dependent diploid species for comparative genetic research. Among them, some species are discarded because of major inconveniences such the lack of nodulation with reference *Bradyrhizobium* strains or the inability to produce seeds in our greenhouse conditions. Based on both efficient nodulation, short flowering time and the ease of seed production, *A. americana* (2n = 20, 600 Mb) and *A. patula* (2n = 20, 270 Mb) appear to be the most promising Nod-dependent diploid species to develop a comparative genetic system with *A. evenia* (2n = 20, 400 Mb). In contrast to *A. evenia*, *A. americana* is nodulated only by non-photosynthetic bradyrhizobia and in this respect, it behaves similarly as to other legumes. This species is widespread in the tropics, hundred of germplasm being available, and it has already been subject to research studies notably to isolate its nodulating *Bradyrhizobium* strains, among which the DOA9 strain [48, 49]. As *A. americana* belongs to the most basal lineage in the *Aeschynomene* phylogeny, it may be representative of the ancestral symbiotic mechanisms found in the genus. On the other hand, *A. patula* has a restricted Malagasy distribution with only one accession available, but it has the interest to be relatively smaller both in plant size and in genome size (actually the smallest diploid genome in the group) making this species the “arabidopsis” of the *Aeschynomene*. Like *A. americana*, this species is efficiently nodulated by non-photosynthetic bradyrhizobia, but it is also compatible with the photosynthetic *nod* gene-containing ORS285 strain. This property makes this species particularly interesting as it allows direct comparisons of mechanisms and pathways between *A. evenia* and *A. patula* without the problem of potential strain effects on symbiotic responses. In addition, when considering the *Aeschynomene* phylogeny, *A. patula* is closer to *A. evenia* than *A. americana* is, and so it may be more suitable to illuminate the changes necessary to switch a Nod-dependent to a Nod-independent process or vice-versa.

## Conclusions

In the present study, we established a comprehensive and robust molecular phylogeny for the genus *Aeschynomene* and related genera, documented with molecular, genomic and nodulation data, in order to unravel the evolutionary history of the whole group. This phylogenetic framework provides support to exploit efficiently the genetic and nodulation diversity encountered in *Aeschynomene* legumes. In the present study, it guided the choice of *A. americana* and *A. patula*, as the two most appropriate Nod-dependent diploid species to develop a comparative genetic system with the Nod-independent *A. evenia* model. Developing sequence resources and functional tools for *A. americana* and/or *A. patula* is now necessary to set up a fully workable comparative *Aeschynomene* system. In the long run, handling such a genetic system will be instrumental in understanding how photosynthetic *Bradyrhizobium* and some *Aeschynomene* species co-evolved and in unravelling the molecular mechanisms of the Nod-independent symbiosis.

## Methods

### Plant material

All the accessions of *Aeschynomene* used in this study, including their geographic origin and collection data are listed in Additional file 1: Table S1 and Additional file 16: Table S4. Seed germination and plant cultivation in the greenhouse were performed as indicated in Arrighi et al. [15]. Phenotypic traits such as the presence of adventitious root primordia and nodules on the stem were directly observed in the glasshouse.

### Nodulation tests

Nodulation tests were carried out using *Bradyrhizobium* strains ORS278 (originally isolated from *A. sensitiva* nodules), ORS285 (originally isolated from *A. afraspera* nodules), ORS285∆*nod* and DOA9 (originally isolated from *A. americana* nodules) [7, 49, 50]. *Bradyrhizobium* strains were cultivated at 34 °C for seven days in Yeast Mannitol (YM) liquid medium supplemented with an antibiotic when necessary [51]. Plant in vitro culture was performed in tubes filled with buffered nodulation medium (BNM) as described in Arrighi et al. [15]. Five-day-old plants were inoculated with 1 mL of bacterial culture with an adjusted OD at 600 nm to 1. Twenty one days after inoculation, six plants were analysed for the presence of root nodules. Nitrogen-fixing activity was estimated on the entire plant by measurement of acetylene reducing activity (ARA) and microscopic observations were performed using a stereo-macroscope (Nikon AZ100, Champigny-sur-Marne, France) as published in Bonaldi et al. [50].

### Molecular methods

Plant genomic DNA was isolated from fresh material using the classical CTAB (Cetyl Trimethyl Ammonium Bromide) extraction method. For herbarium material, the method was adapted by increasing the length of the incubation (90 min), centrifugation (20 min) and precipitation (15 min) steps. The nuclear ribosomal internal transcribed spacer region (ITS), the chloroplast *matK* gene and four low-copy nuclear genes (*CYP1*, *eiF1α*, *SuSy*, and *TIP1;1*) previously identified in the *A. evenia* and *A. afraspera* transcriptomes were used for phylogenetic analyses [27, 28]. The genes were PCR-amplified, cloned and sequenced as described in Arrighi et al. [27] (Additional file 2: Table S2). For genomic DNA extracted from herbarium specimens, a battery of primers was developed to amplify the different genes in overlapping fragments as short as 250 bp (Additional file 2: Table S2). The DNA sequences generated in this study were deposited in GenBank (Additional file 3: Table S3).

### Phylogenetic analyses and traits mapping

Sequences were aligned using MAFFT (*−-localpair –maxiterate 1000*; [52]). Phylogenetic reconstructions were performed for each gene as well as for concatenated datasets under a Bayesian approach using Phylobayes 4.1b [53] and the site-heterogeneous CAT+F81 + Γ4 evolution model. For each analysis, two independent chains were run for 10,000 Phylobayes cycles with a 50% burn-in. Ancestral states reconstruction was done through stochastic character mapping using the Phytools R package [54] running 10 simulations for each character.

### Species networks and hybridizations

To test if the phylogeny obtained by concatenating the four low-copy nuclear genes (*CYP1*, *eiF1α*, *SuSy*, and *TIP1;1*) was most likely obtained by gene duplications followed by differential losses or by a combination of duplications, losses coupled with one or several allopolyploidy events involving *A. patula* and *Soemmeringia semperflorens*, the method presented in [55] was used. In short, this method computes a reconciliation score by comparing a phylogenetic network and one or several gene trees. The method allows allopolyploidy events at hybridization nodes while all other nodes of the network are associated to speciation events; meanwhile, duplication and loss events are allowed at a cost (here, arbitrarily fixed to 1) on all nodes of the gene tree.

Thus, the set of 4 nuclear gene trees was used to score different phylogenetic networks corresponding to four different potential evolutionary histories. Two alternative networks with no reticulation corresponding to the two topologies obtained either with the group A (T1) or group B (T2) served to evaluate a no-allopolyploidisation hypothesis. The topology yielding the best score (T2) served to generate and compare all phylogenetic networks with one or two hybridization nodes, involving *A. patula* and/or *S. semperflorens*, to test successively a one-allopolyploidisation scenario (N1-best) and a two-allopolyploidisation evolutionary scenario (N2-best).

### GBS analysis

A GBS library was constructed based on a protocol described [56]. For each sample, a total of 150 ng of genomic DNA was digested using the two-enzyme system, PstI (rare cutter) and Mse (common cutter) (New England Biolabs, Hitchin, UK), by incubating at 37 °C for 2 h. The ligation reaction was performed using the T4 DNA ligase enzyme (New England Biolabs, Hitchin, UK) at 22 °C for 30 min and the ligase was inactivated at 65 °C for 30 min. Ligated samples were pooled and PCR-amplified using the Illumina Primer 1 (barcoded adapter with PstI overhang) and Illumina Primer 2 (common Y-adapter). The library was sequenced on an Illumina HiSeq 3000 (1 × 150 pb) (at the Get-PlaGe platform in Toulouse, France).

The raw sequence data were processed in the same way as in the study described in [57]. SNP calling from the raw Illumina reads was performed using the custom python pipeline VcfHunter (available at https://github.com/SouthGreenPlatform/VcfHunter/) (Guillaume Martin, CIRAD, France). For all samples, these sequence tags were aligned to the *A. evenia* 1.0 reference genome (JF Arrighi, unpublished data). The SNP results from all the samples were converted into one large file in VCF format and the polymorphism data were subsequently analyzed using the web-based application SNiPlay3 [58]. First, the SNP data were treated separately for each species and filtered out to remove SNP with more than 10% missing data as well as those with a minor allele frequency (MAF) 0.01 using integrated VCFtools. Second, an overall representation of the species diversity structures was obtained by making use of the PLINK software as implemented in SNiPlay3. This software is based on the multidimensional-scaling (MSD) method to produce two-dimensional plots.

### Genome size estimation and chromosome counting

Genome sizes were measured by flow cytometry using leaf material as described in Arrighi et al. [15]. Genome size estimations resulted from measurements of three plants per accession and *Lycopersicum esculentum* (Solanaceae) cv “Roma” (2C = 1.99 pg) was used as the internal standard. The 1C value was calculated and the conversion factor 1 pg DNA = 978 Mb was used to express it in Mb/1C. To count chromosome number, metaphasic chromosomes were prepared from root-tips, spread on slides, stained with 4′,6-diamidino-2-phenylindole (DAPI) and their image captured with a fluorescent microscope as detailed in Arrighi et al. [15] .

## Additional files

Additional file 1: **Table S1.** Accessions used for the phylogeny of the genus *Aeschynomene* and related genera, their origin and characteristics. (PPTX 143 kb)

Additional file 2: **Table S2.** Primers used for gene amplification and sequencing. (PPTX 134 kb)

Additional file 3: **Table S3.** GenBank numbers for the sequences used in the phylogenetic analyses. (PPTX 149 kb)

Additional file 4: **Figure S1.** *matK* phylogeny of the genus *Aeschynomene* and allied genera. Bayesian phylogenetic reconstruction obtained using the chloroplastic *matK* gene. Numbers at branches are posterior probability. (PPTX 133 kb)

Additional file 5: **Figure S2.** *ITS* phylogeny of the genus *Aeschynomene* and allied genera. Bayesian phylogenetic reconstruction obtained using the Internal Transcribed Spacer (*ITS*) sequence. Numbers at branches are posterior probability. (PPTX 134 kb)

Additional file 6: **Figure S3.** Chromosome numbers in *Aeschynomene* species. Root tip metaphase chromosomes stained in blue with DAPI (4′,6-diamidino-2-phenylindole). Chromosome numbers are indicated in brackets. Scale bars: 5 μm. (PPTX 135 kb)

Additional file 7: **Figure S4.** Chromosome numbers in species of *Aeschynomene* related genera. Root tip metaphase chromosomes stained in blue with DAPI (4′,6-diamidino-2-phenylindole). Chromosome counts are indicated in brackets. Scale bars: 5 μm. (PPTX 57 kb)

Additional file 8: **Figure S5.** Phylogenetic trees based on nuclear low-copy genes. Bayesian phylogenetic reconstructions obtained for the *CYP1*, *eif1a*, *SuSy* and *TIP1;1* genes. Diploid species (2n = 20) are in blue, polyploid species (2n ≥ 28) in black excepted *A. afraspera* for which the A and B gene copies are distinguished in red and green respectively. -A, −A1, −A2, -B, -B1 and -B2 indicated the different copies found. Putative A and B subgenomes of the polyploid taxa are delineated by red and green boxes in dashed lines, respectively. Numbers at branches represent posterior probability. (PPTX 56 kb)

Additional file 9: **Figure S6.** Ancestral state reconstruction of ploidy levels in the genus *Aeschynomene* and allied genera. Ancestral state reconstruction was estimated in SIMMAP software using the 50% majority-rule topology obtained by Bayesian analysis of the combined *ITS* + *matK* sequences. Ploidy levels are indicated by different colors. Unknown ploidy levels are denoted by a dash. (PPTX 3568 kb)

Additional file 10: **Figure S7.** Phylogenetic networks based on the four nuclear *CYP1*, *eif1a*, *SuSy* and *TIP1;1* genes. (a) No-allopolyploidisation hypothesis (T1) based on the concatenated gene tree obtained taking into account the group A (Fig. 2b). (b) No-allopolyploidisation hypothesis (T2) based on the concatenated gene tree obtained taking into account the group B (Fig. 2b). (c) One-allopolyploidisation hypothesis (N1-best). (d) Two-allopolyploidisation hypothesis (N2-best). Blue lines indicate reticulations while other nods of the network are associated to speciation events. Scores obtained for the different phylogenetic networks are indicated. (PPTX 2589 kb)

Additional file 11: **Figure S8.** Ancestral state reconstruction of adventive root primordia in the genus *Aeschynomene* and allied genera. Ancestral state reconstruction was estimated in SIMMAP software using the 50% majority-rule topology obtained by Bayesian analysis of the combined *ITS* + *matK* sequences. Data on the adventitious root primordia come from the present analysis and pertinent previously published data. Presence or not of adventitious root primordia is indicated by different colors. (PPTX 96 kb)

Additional file 12: **Figure S9.** Ancestral state reconstruction of ecological habit in the genus *Aeschynomene* and allied genera. Ancestral state reconstruction was estimated in SIMMAP software using the 50% majority-rule topology obtained by Bayesian analysis of the combined *ITS* + *matK* sequences. Data on the species ecology come from pertinent previously published data. Ecological habits are indicated by different colors. (PPTX 135 kb)

Additional file 13: **Figure S10.** Ancestral state reconstruction of the aerial stem nodulation ability in the genus *Aeschynomene* and allied genera. Ancestral state reconstruction was estimated in SIMMAP software using the 50% majority-rule topology obtained by Bayesian analysis of the combined *ITS* + *matK* sequences. Data on the occurrence of stem nodulation come from pertinent previously published data. Occurrence or not of stem nodulation is indicated by different colors. (XLSX 15 kb)

Additional file 14: **Figure S11.** Ancestral state reconstruction of the ability to nodulate with the photosynthetic *Bradyrhizobium* strains in the genus *Aeschynomene* and allied genera. Ancestral state reconstruction was estimated in SIMMAP software using the 50% majority-rule topology obtained by Bayesian analysis of the combined *ITS* + *matK* sequences. Data on nodulation with photosynthetic *Bradyrhizobium* strains come from the present analysis and pertinent previously published data. Nodulation with photosynthetic *Bradyrhizobium* strains is considered positive only if reported as occurring naturally or being efficient in vitro. (XLSX 13 kb)

Additional file 15: **Figure S12.** Ancestral state reconstruction of the ability to nodulate with the photosynthetic *Bradyrhizobium* strain ORS278 in the genus *Aeschynomene* and allied genera. Ancestral state reconstruction was estimated in SIMMAP software using the 50% majority-rule topology obtained by Bayesian analysis of the combined *ITS* + *matK* sequences. Data on nodulation with ORS278 come from the present analysis and pertinent previously published data. Ability or not to nodulate with ORS278 is indicated by different colors. (XLSX 16 kb) (XLSX 11 kb)

Additional file 16: **Table S4.** *A. americana* and *A. villosa* accessions used for the GBS analysis, their origin and characteristics. (XLSX 16 kb)

## Abbreviations

ARA: Acetylene reduction assay
BNM: Buffered nodulation medium
BRH: Clade containing *Aeschynomene* subgenera *Bakerophyton* and *Rueppellia* together with the genus *Humularia*
CI: Cross-inoculation
DAPI: 4′,6-diamidino-2-phenylindole
dpi: Days-post-germination
GBS: Genotyping-by-sequencing
MSD: Multidimensional-scaling
PP: Posterior probability
SNP: Single nucleotide polymorphism
T3SS: Type III secretion system
YM: Yeast medium
