# Supplementary material for: A phylogenetic framework of the legume genus Aeschynomene for comparative genetic analysis of the Nod-dependent and Nod-independent symbioses
Source: BMC Plant Biol. 2018 Dec 5;18:333. doi: 10.1186/s12870-018-1567-z (PMC6282307; doi:10.1186/s12870-018-1567-z)
Supplement: Supplementary file 1 — Table S1. Accessions used for the phylogeny of the genus Aeschynomene and related genera, their origin and characteristics. (PPTX 143 kb) [file 12870_2018_1567_MOESM1_ESM.pptx]

## Slide 1
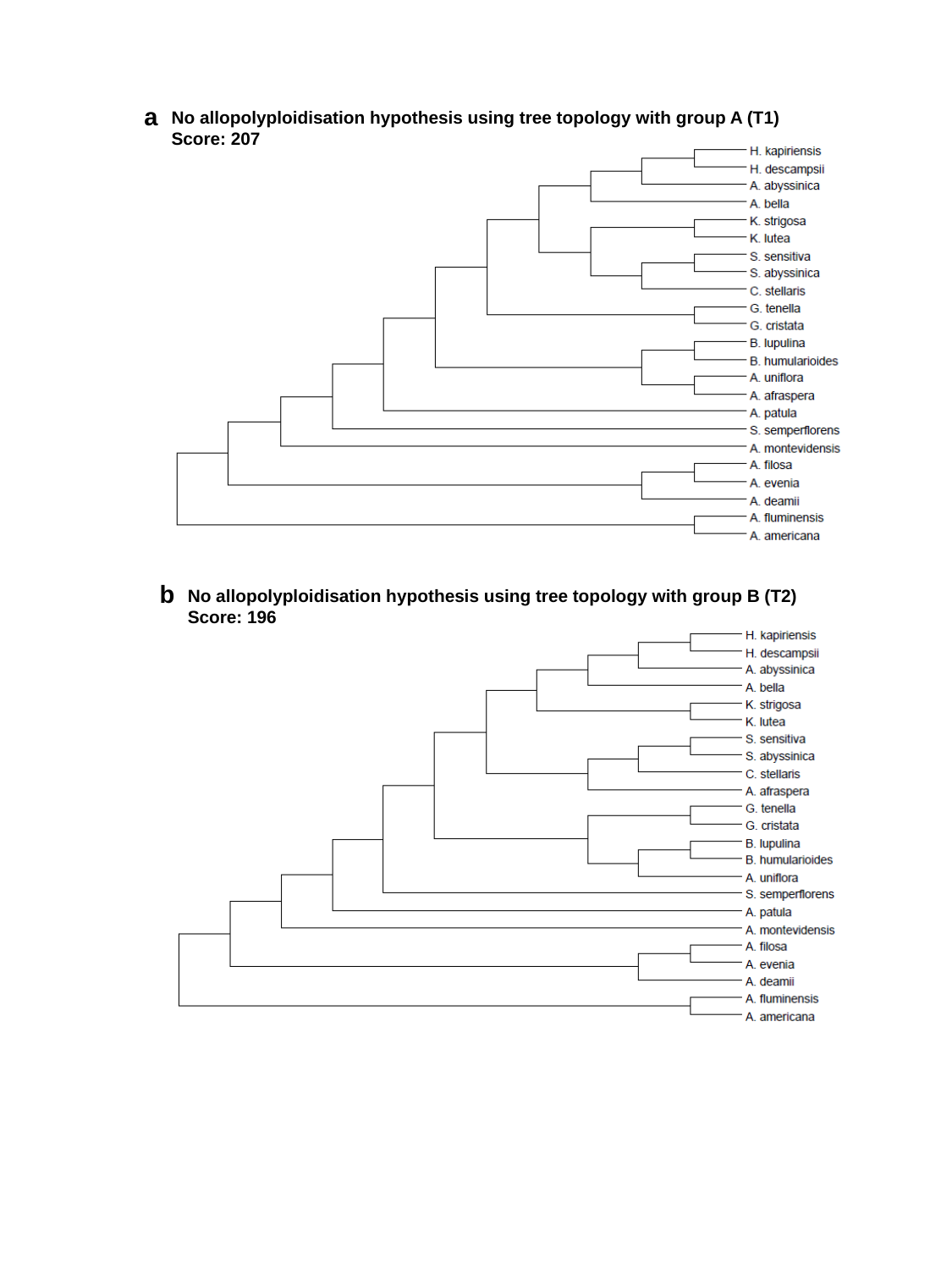

a
No allopolyploidisation hypothesis using tree topology with group A (T1)
Score: 207
b
No allopolyploidisation hypothesis using tree topology with group B (T2)
Score: 196

## Slide 2
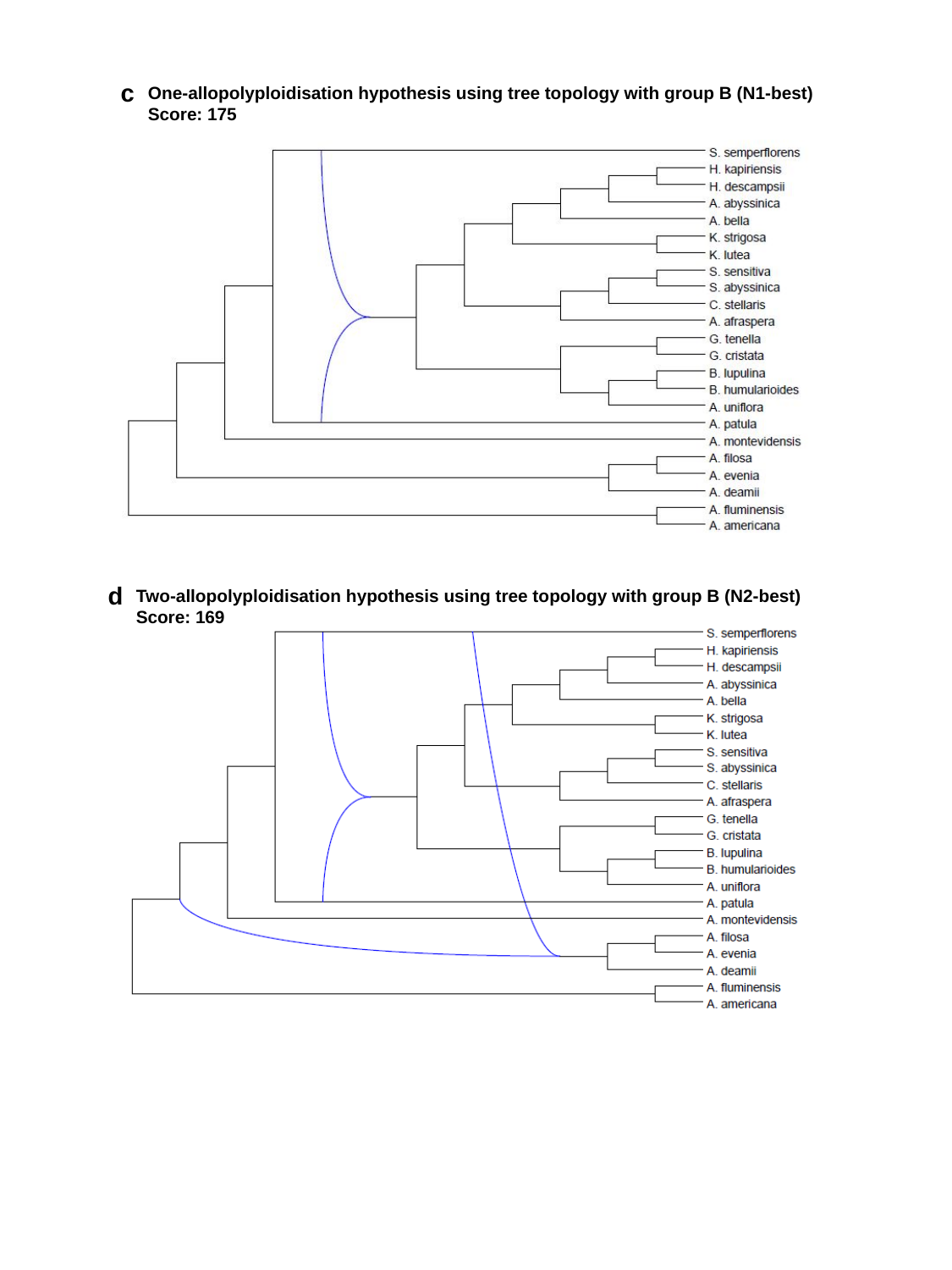

c
One-allopolyploidisation hypothesis using tree topology with group B (N1-best)
Score: 175
d
Two-allopolyploidisation hypothesis using tree topology with group B (N2-best)
Score: 169
