# Supplementary figures and images for: A phylogenetic framework of the legume genus Aeschynomene for comparative genetic analysis of the Nod-dependent and Nod-independent symbioses
Source: BMC Plant Biol. 2018 Dec 5;18:333. doi: 10.1186/s12870-018-1567-z (PMC6282307; doi:10.1186/s12870-018-1567-z)

## Slide 1
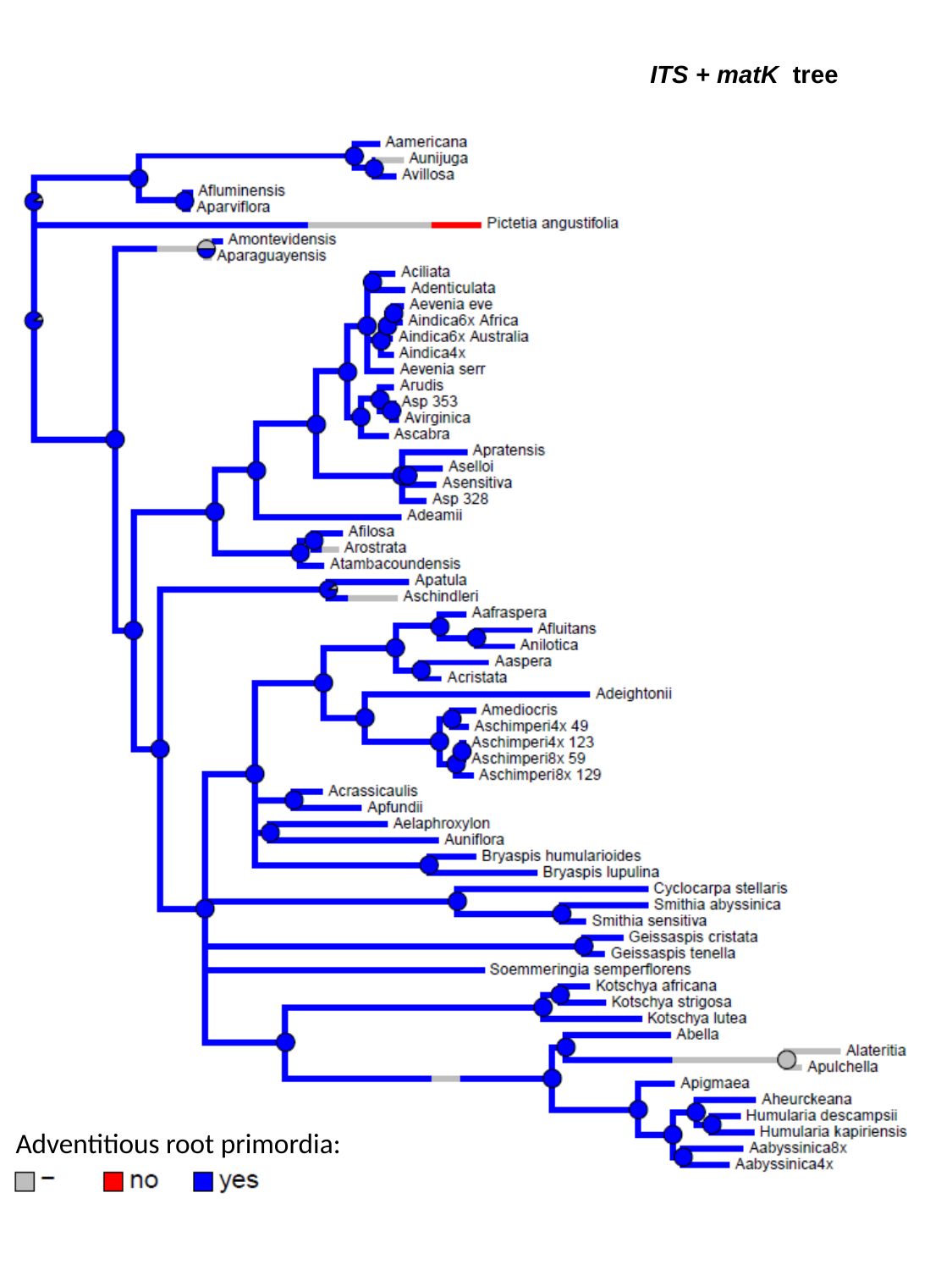

ITS + matK tree
Adventitious root primordia:

Supplement: Supplementary file 2 — Table S2. Primers used for gene amplification and sequencing. (PPTX 134 kb) [file 12870_2018_1567_MOESM2_ESM.pptx]

## Slide 1
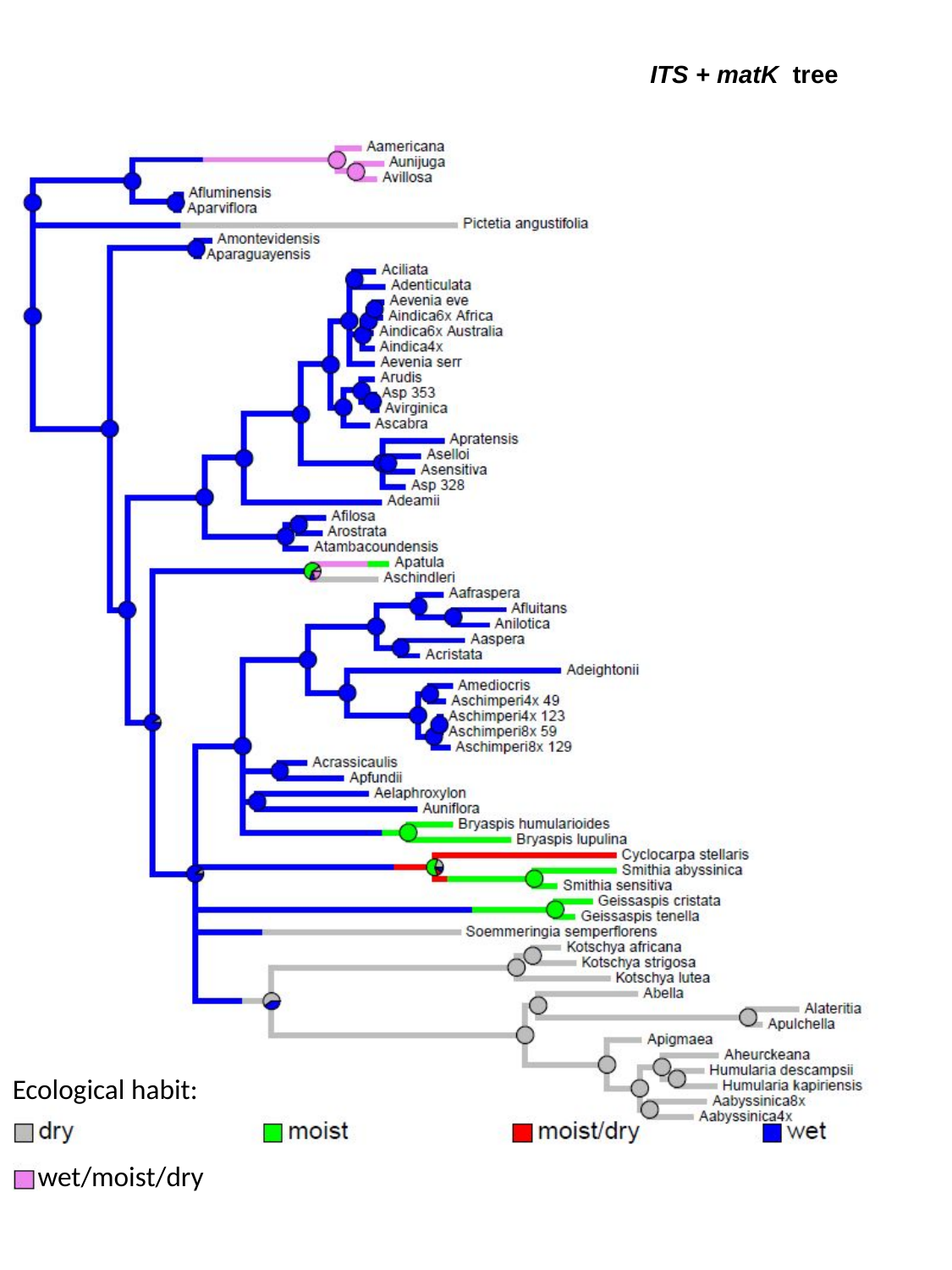

ITS + matK tree
Ecological habit:
wet/moist/dry

Supplement: Supplementary file 3 — Table S3. GenBank numbers for the sequences used in the phylogenetic analyses. (PPTX 149 kb) [file 12870_2018_1567_MOESM3_ESM.pptx]

## Slide 1
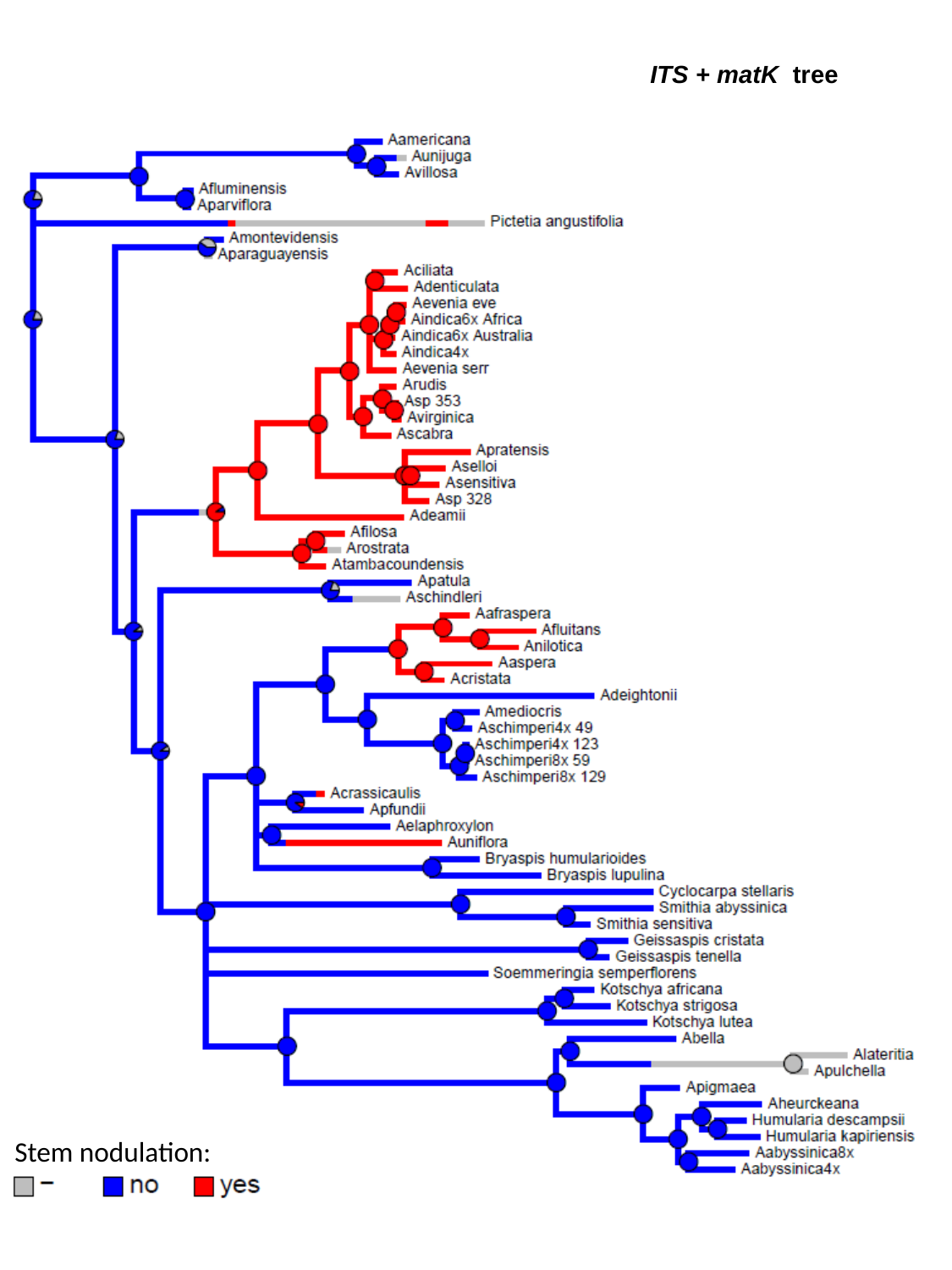

ITS + matK tree
Stem nodulation:

Supplement: Supplementary file 4 — Figure S1. matK phylogeny of the genus Aeschynomene and allied genera. Bayesian phylogenetic reconstruction obtained using the chloroplastic matK gene. Numbers at branches are posterior probability. (PPTX 133 kb) [file 12870_2018_1567_MOESM4_ESM.pptx]

## Slide 1
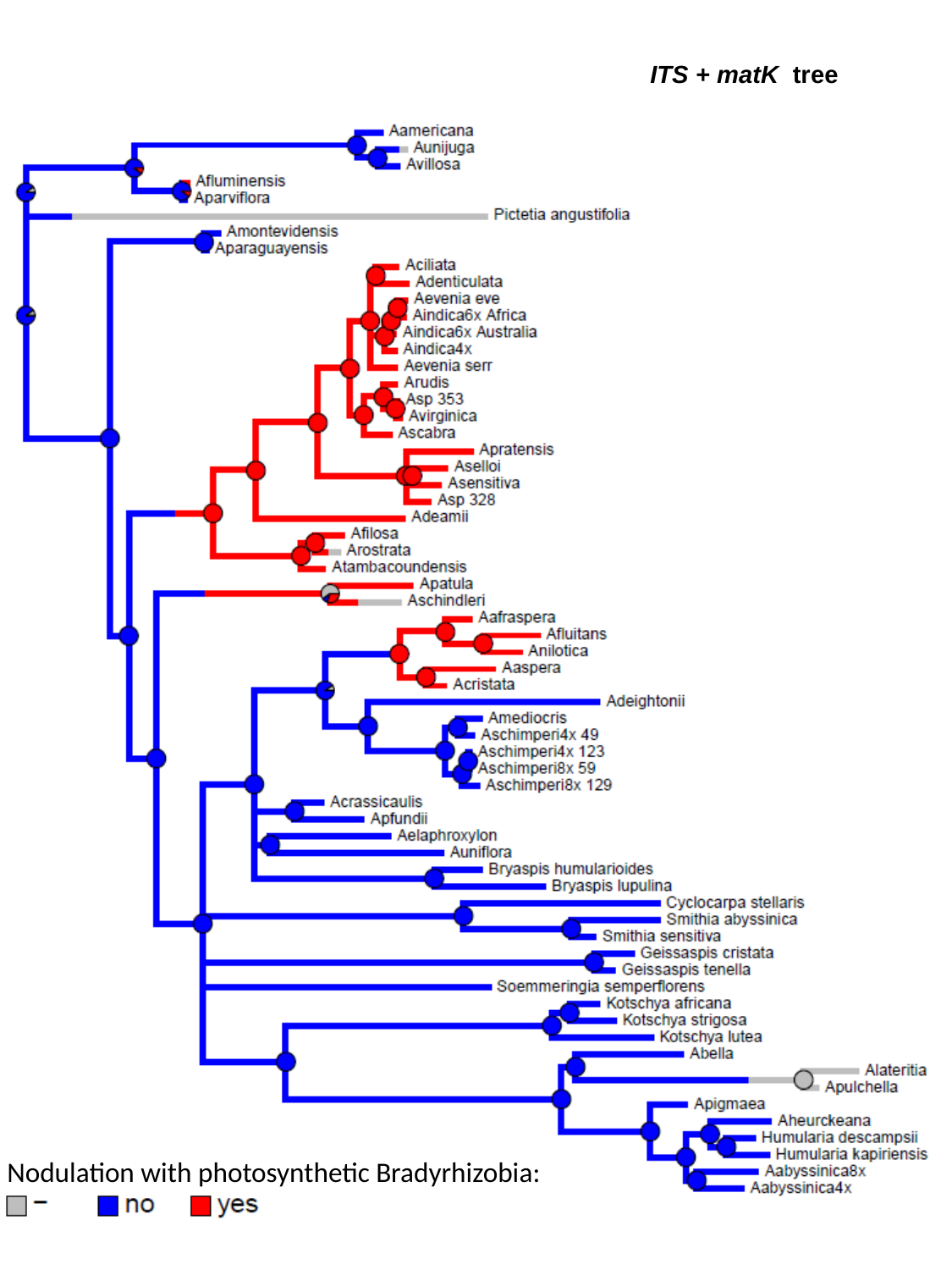

ITS + matK tree
Nodulation with photosynthetic Bradyrhizobia:

Supplement: Supplementary file 5 — Figure S2. ITS phylogeny of the genus Aeschynomene and allied genera. Bayesian phylogenetic reconstruction obtained using the Internal Transcribed Spacer (ITS) sequence. Numbers at branches are posterior probability. (PPTX 134 kb) [file 12870_2018_1567_MOESM5_ESM.pptx]

## Slide 1
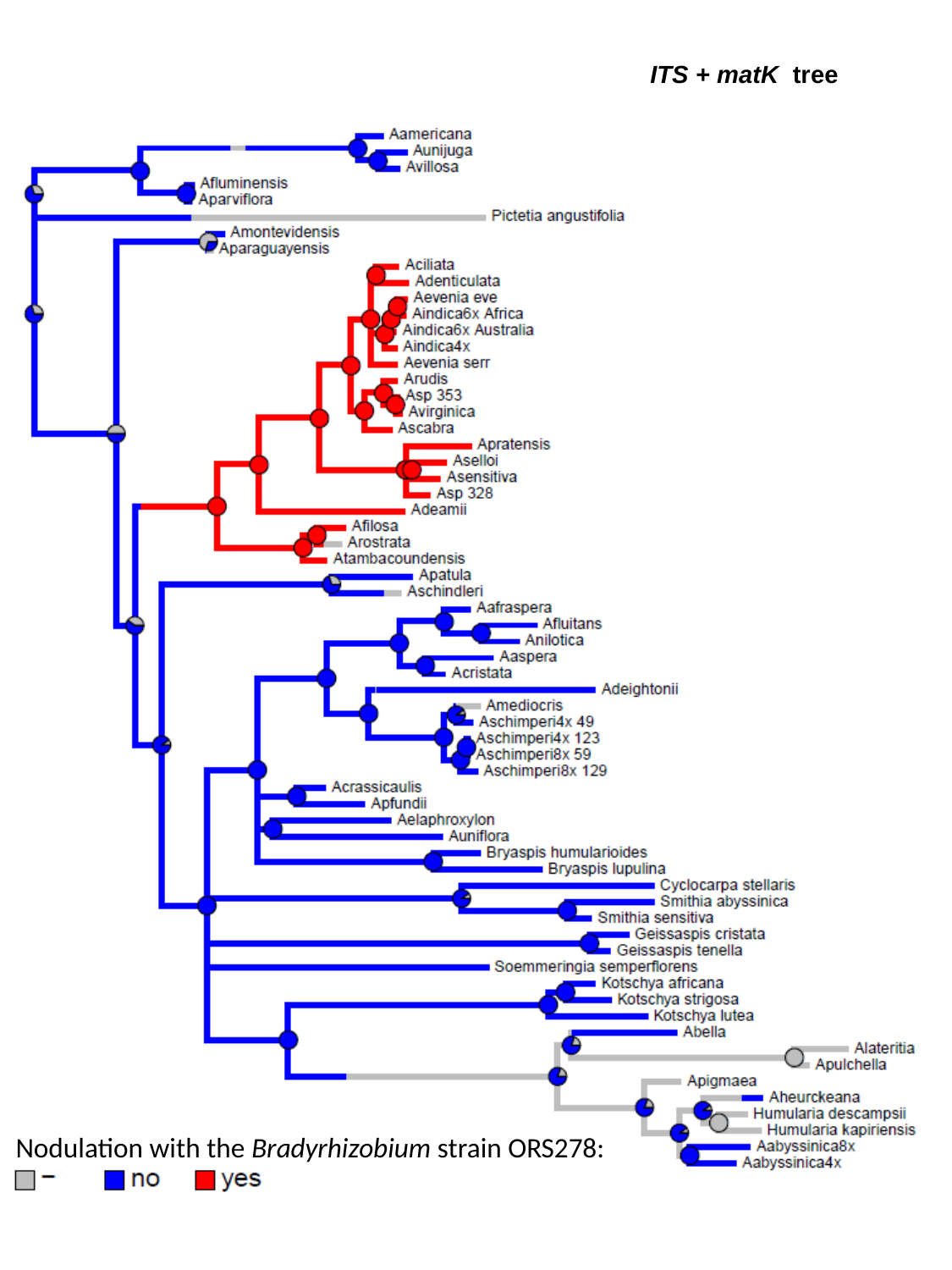

ITS + matK tree
Nodulation with the Bradyrhizobium strain ORS278:

Supplement: Supplementary file 6 — Figure S3. Chromosome numbers in Aeschynomene species. Root tip metaphase chromosomes stained in blue with DAPI (4′,6-diamidino-2-phenylindole). Chromosome numbers are indicated in brackets. Scale bars: 5 μm. (PPTX 135 kb) [file 12870_2018_1567_MOESM6_ESM.pptx]

## Slide 1
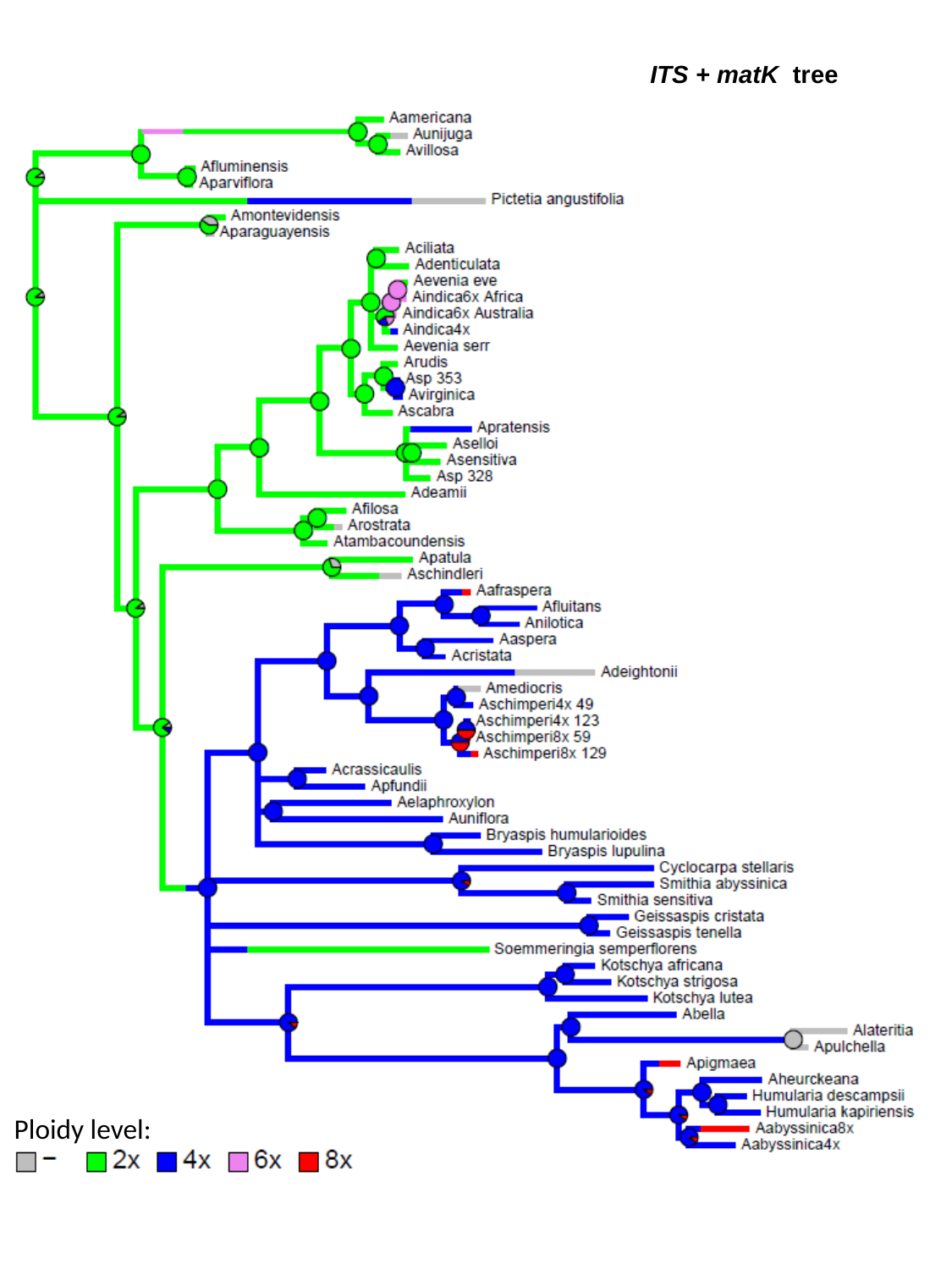

ITS + matK tree
Ploidy level:

Supplement: Supplementary file 12 — Figure S9. Ancestral state reconstruction of ecological habit in the genus Aeschynomene and allied genera. Ancestral state reconstruction was estimated in SIMMAP software using the 50% majority-rule topology obtained by Bayesian analysis of the combined ITS + matK sequences. Data on the species ecology come from pertinent previously published data. Ecological habits are indicated by different colors. (PPTX 135 kb) [file 12870_2018_1567_MOESM12_ESM.pptx]
