# Supplementary material for: A phylogenetic framework of the legume genus Aeschynomene for comparative genetic analysis of the Nod-dependent and Nod-independent symbioses
Source: BMC Plant Biol. 2018 Dec 5;18:333. doi: 10.1186/s12870-018-1567-z (PMC6282307; doi:10.1186/s12870-018-1567-z)
Supplement: Supplementary file 7 — Figure S4. Chromosome numbers in species of Aeschynomene related genera. Root tip metaphase chromosomes stained in blue with DAPI (4′,6-diamidino-2-phenylindole). Chromosome counts are indicated in brackets. Scale bars: 5 μm. (PPTX 57 kb) [file 12870_2018_1567_MOESM7_ESM.pptx]

## Slide 1
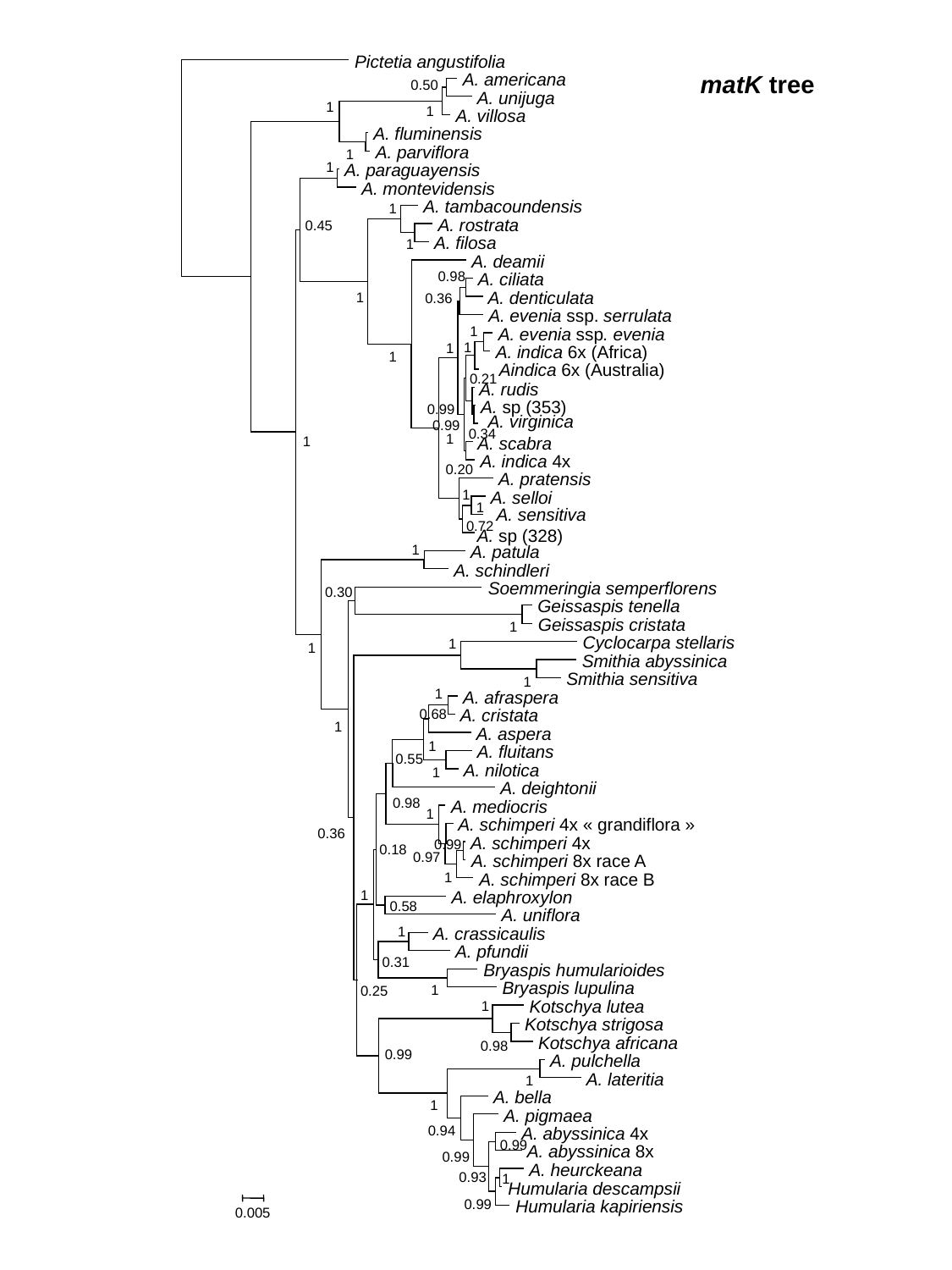

Pictetia angustifolia
matK tree
 A. americana
0.50
 A. unijuga
1
1
 A. villosa
 A. fluminensis
 A. parviflora
1
1
 A. paraguayensis
 A. montevidensis
 A. tambacoundensis
1
 A. rostrata
0.45
 A. filosa
1
 A. deamii
0.98
 A. ciliata
 A. denticulata
1
0.36
 A. evenia ssp. serrulata
1
 A. evenia ssp. evenia
1
1
 A. indica 6x (Africa)
1
 Aindica 6x (Australia)
0.21
 A. rudis
 A. sp (353)
0.99
 A. virginica
0.99
0.34
1
 A. scabra
1
 A. indica 4x
0.20
 A. pratensis
1
 A. selloi
1
 A. sensitiva
0.72
 A. sp (328)
 A. patula
1
 A. schindleri
 Soemmeringia semperflorens
0.30
 Geissaspis tenella
 Geissaspis cristata
1
 Cyclocarpa stellaris
1
1
 Smithia abyssinica
 Smithia sensitiva
1
1
 A. afraspera
 A. cristata
0.68
1
 A. aspera
1
 A. fluitans
0.55
 A. nilotica
1
 A. deightonii
0.98
 A. mediocris
1
 A. schimperi 4x « grandiflora »
0.36
 A. schimperi 4x
0.99
0.18
0.97
 A. schimperi 8x race A
 A. schimperi 8x race B
1
 A. elaphroxylon
1
0.58
 A. uniflora
 A. crassicaulis
1
 A. pfundii
0.31
 Bryaspis humularioides
 Bryaspis lupulina
1
0.25
 Kotschya lutea
1
 Kotschya strigosa
 Kotschya africana
0.98
0.99
 A. pulchella
 A. lateritia
1
 A. bella
1
 A. pigmaea
0.94
 A. abyssinica 4x
0.99
 A. abyssinica 8x
0.99
 A. heurckeana
0.93
1
 Humularia descampsii
 Humularia kapiriensis
0.99
0.005
