# Supplementary material for: A phylogenetic framework of the legume genus Aeschynomene for comparative genetic analysis of the Nod-dependent and Nod-independent symbioses
Source: BMC Plant Biol. 2018 Dec 5;18:333. doi: 10.1186/s12870-018-1567-z (PMC6282307; doi:10.1186/s12870-018-1567-z)
Supplement: Supplementary file 8 — Figure S5. Phylogenetic trees based on nuclear low-copy genes. Bayesian phylogenetic reconstructions obtained for the CYP1, eif1a, SuSy and TIP1;1 genes. Diploid species (2n = 20) are in blue, polyploid species (2n ≥ 28) in black excepted A. afraspera for which the A and B gene copies are distinguished in red and green respectively. -A, −A1, −A2, -B, -B1 and -B2 indicated the different copies found. Putative A and B subgenomes of the polyploid taxa are delineated by red and green boxes in dashed lines, respectively. Numbers at branches represent posterior probability. (PPTX 56 kb) [file 12870_2018_1567_MOESM8_ESM.pptx]

## Slide 1
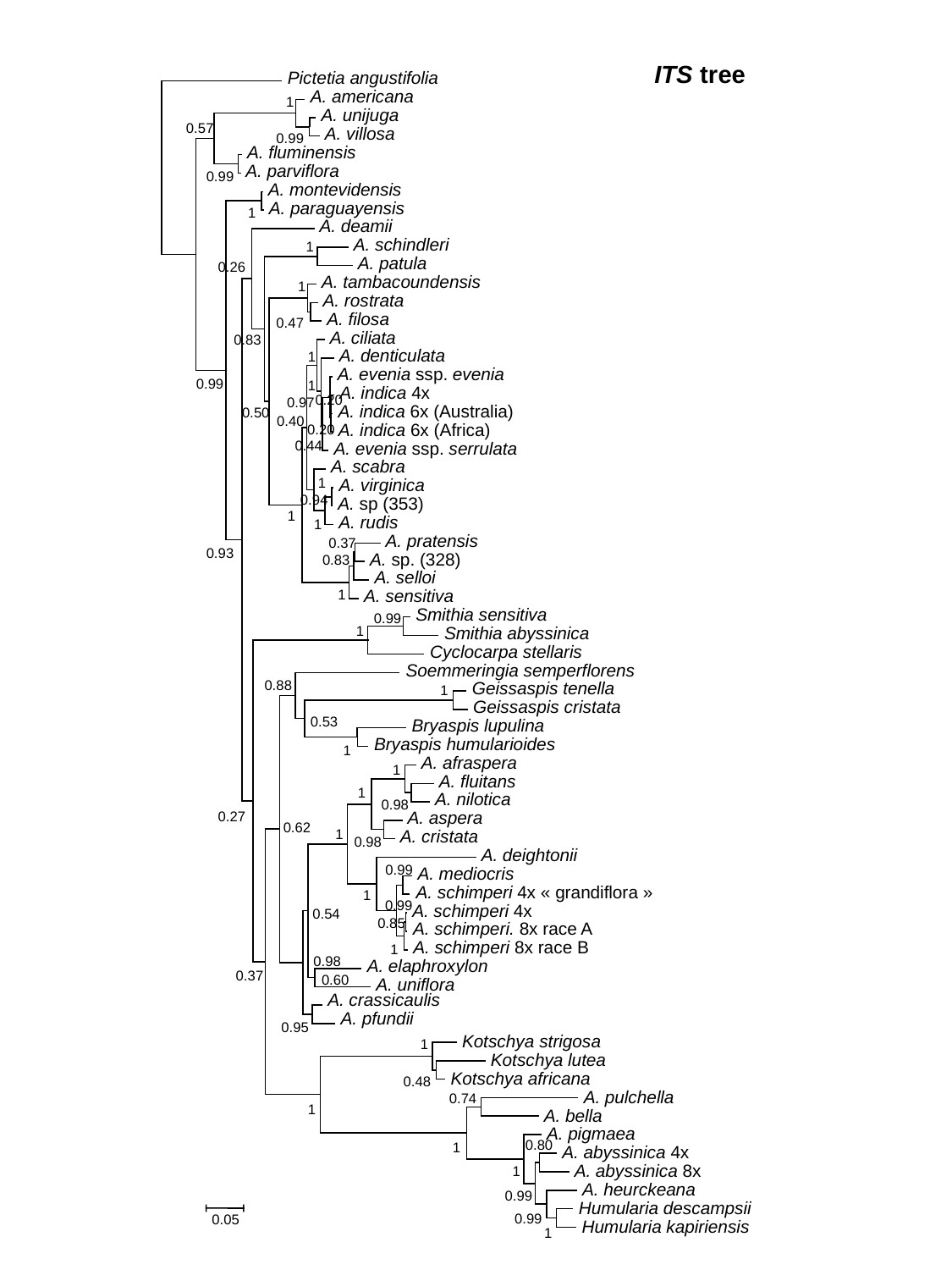

ITS tree
 Pictetia angustifolia
 A. americana
1
 A. unijuga
0.57
 A. villosa
0.99
 A. fluminensis
 A. parviflora
0.99
 A. montevidensis
 A. paraguayensis
1
 A. deamii
 A. schindleri
1
 A. patula
0.26
 A. tambacoundensis
1
 A. rostrata
 A. filosa
0.47
 A. ciliata
0.83
 A. denticulata
1
 A. evenia ssp. evenia
0.99
1
 A. indica 4x
0.20
0.97
 A. indica 6x (Australia)
0.50
0.40
 A. indica 6x (Africa)
0.20
0.44
 A. evenia ssp. serrulata
 A. scabra
1
 A. virginica
0.94
 A. sp (353)
1
 A. rudis
1
 A. pratensis
0.37
0.93
 A. sp. (328)
0.83
 A. selloi
 A. sensitiva
1
 Smithia sensitiva
0.99
 Smithia abyssinica
1
 Cyclocarpa stellaris
 Soemmeringia semperflorens
0.88
 Geissaspis tenella
1
 Geissaspis cristata
0.53
 Bryaspis lupulina
 Bryaspis humularioides
1
 A. afraspera
1
 A. fluitans
1
 A. nilotica
0.98
 A. aspera
0.27
0.62
1
 A. cristata
0.98
 A. deightonii
0.99
 A. mediocris
 A. schimperi 4x « grandiflora »
1
0.99
 A. schimperi 4x
0.54
0.85
 A. schimperi. 8x race A
 A. schimperi 8x race B
1
0.98
 A. elaphroxylon
0.37
0.60
 A. uniflora
 A. crassicaulis
 A. pfundii
0.95
 Kotschya strigosa
1
 Kotschya lutea
 Kotschya africana
0.48
 A. pulchella
0.74
1
 A. bella
 A. pigmaea
0.80
1
 A. abyssinica 4x
 A. abyssinica 8x
1
 A. heurckeana
0.99
 Humularia descampsii
0.99
0.05
 Humularia kapiriensis
1
