# Supplementary material for: A phylogenetic framework of the legume genus Aeschynomene for comparative genetic analysis of the Nod-dependent and Nod-independent symbioses
Source: BMC Plant Biol. 2018 Dec 5;18:333. doi: 10.1186/s12870-018-1567-z (PMC6282307; doi:10.1186/s12870-018-1567-z)
Supplement: Supplementary file 9 — Figure S6. Ancestral state reconstruction of ploidy levels in the genus Aeschynomene and allied genera. Ancestral state reconstruction was estimated in SIMMAP software using the 50% majority-rule topology obtained by Bayesian analysis of the combined ITS + matK sequences. Ploidy levels are indicated by different colors. Unknown ploidy levels are denoted by a dash. (PPTX 3568 kb) [file 12870_2018_1567_MOESM9_ESM.pptx]

## Slide 1
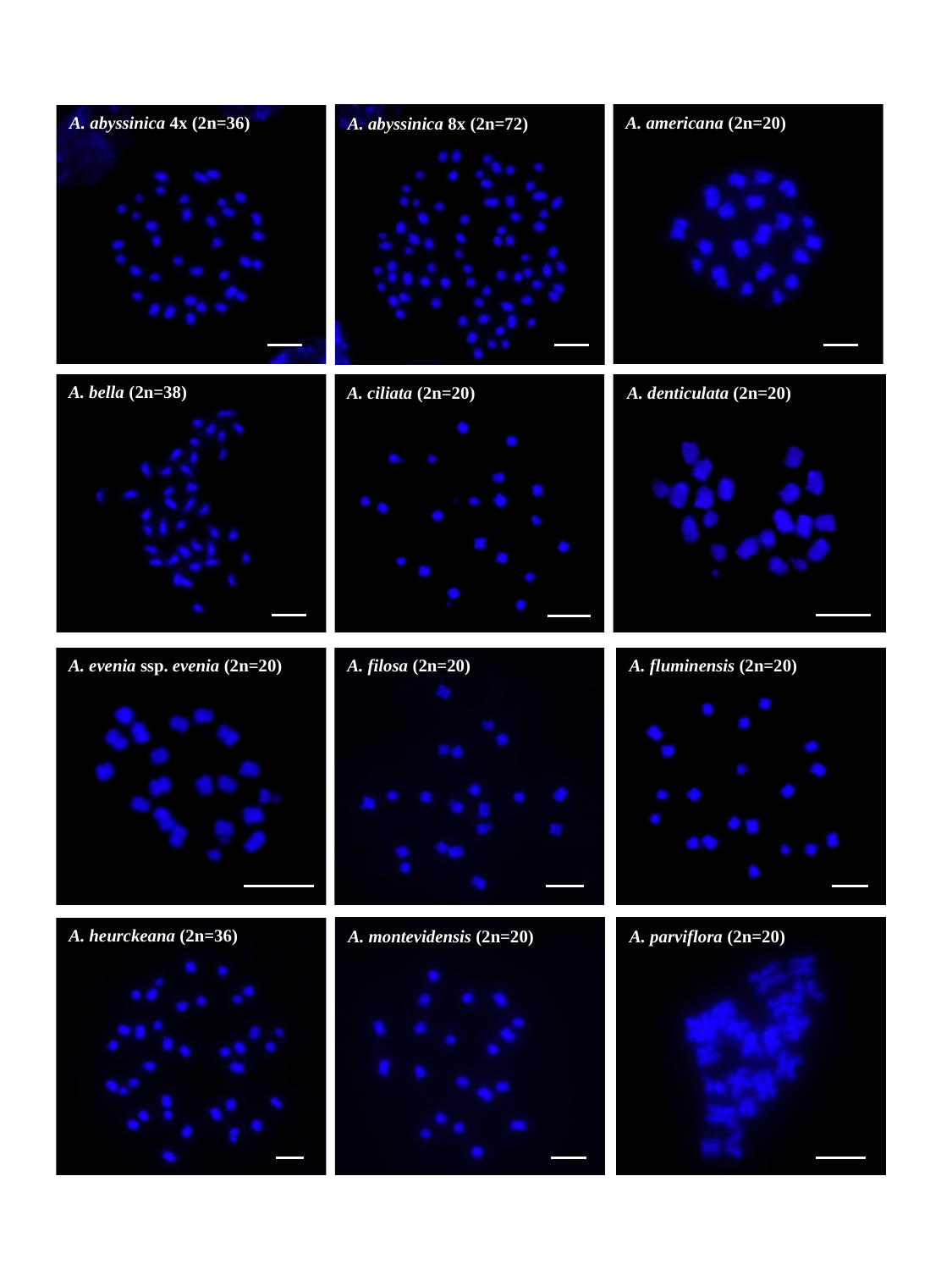

A. americana (2n=20)
A. abyssinica 4x (2n=36)
A. abyssinica 8x (2n=72)
A. bella (2n=38)
A. ciliata (2n=20)
A. denticulata (2n=20)
A. fluminensis (2n=20)
A. evenia ssp. evenia (2n=20)
A. filosa (2n=20)
A. heurckeana (2n=36)
A. montevidensis (2n=20)
A. parviflora (2n=20)

## Slide 2
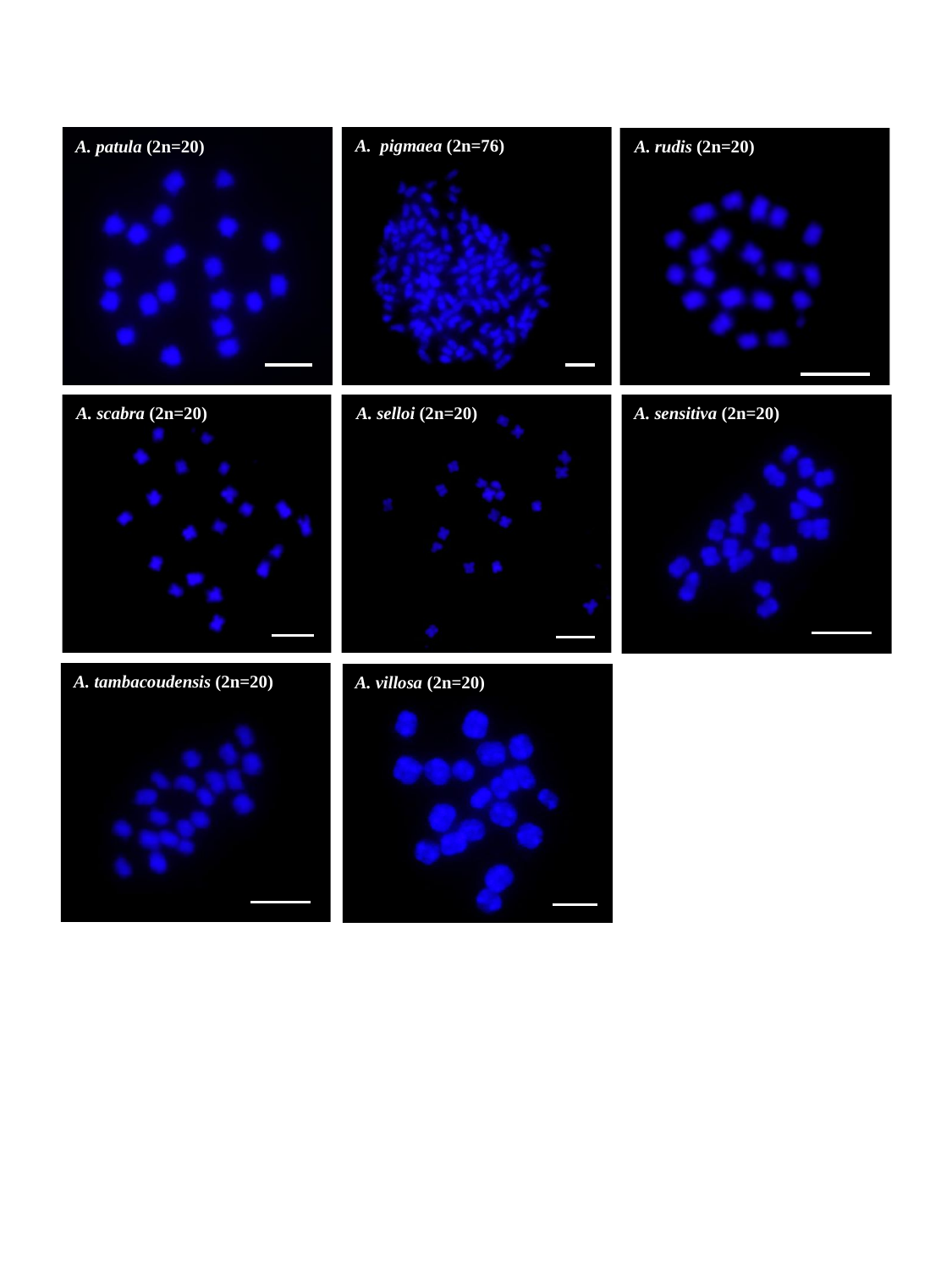

A. pigmaea (2n=76)
A. patula (2n=20)
A. rudis (2n=20)
A. sensitiva (2n=20)
A. selloi (2n=20)
A. scabra (2n=20)
A. tambacoudensis (2n=20)
A. villosa (2n=20)
