# Supplementary material for: A phylogenetic framework of the legume genus Aeschynomene for comparative genetic analysis of the Nod-dependent and Nod-independent symbioses
Source: BMC Plant Biol. 2018 Dec 5;18:333. doi: 10.1186/s12870-018-1567-z (PMC6282307; doi:10.1186/s12870-018-1567-z)
Supplement: Supplementary file 10 — Figure S7. Phylogenetic networks based on the four nuclear CYP1, eif1a, SuSy and TIP1;1 genes. (a) No-allopolyploidisation hypothesis (T1) based on the concatenated gene tree obtained taking into account the group A (Fig. 2b). (b) No-allopolyploidisation hypothesis (T2) based on the concatenated gene tree obtained taking into account the group B (Fig. 2b). (c) One-allopolyploidisation hypothesis (N1-best). (d) Two-allopolyploidisation hypothesis (N2-best). Blue lines indicate reticulations while other nods of the network are associated to speciation events. Scores obtained for the different phylogenetic networks are indicated. (PPTX 2589 kb) [file 12870_2018_1567_MOESM10_ESM.pptx]

## Slide 1
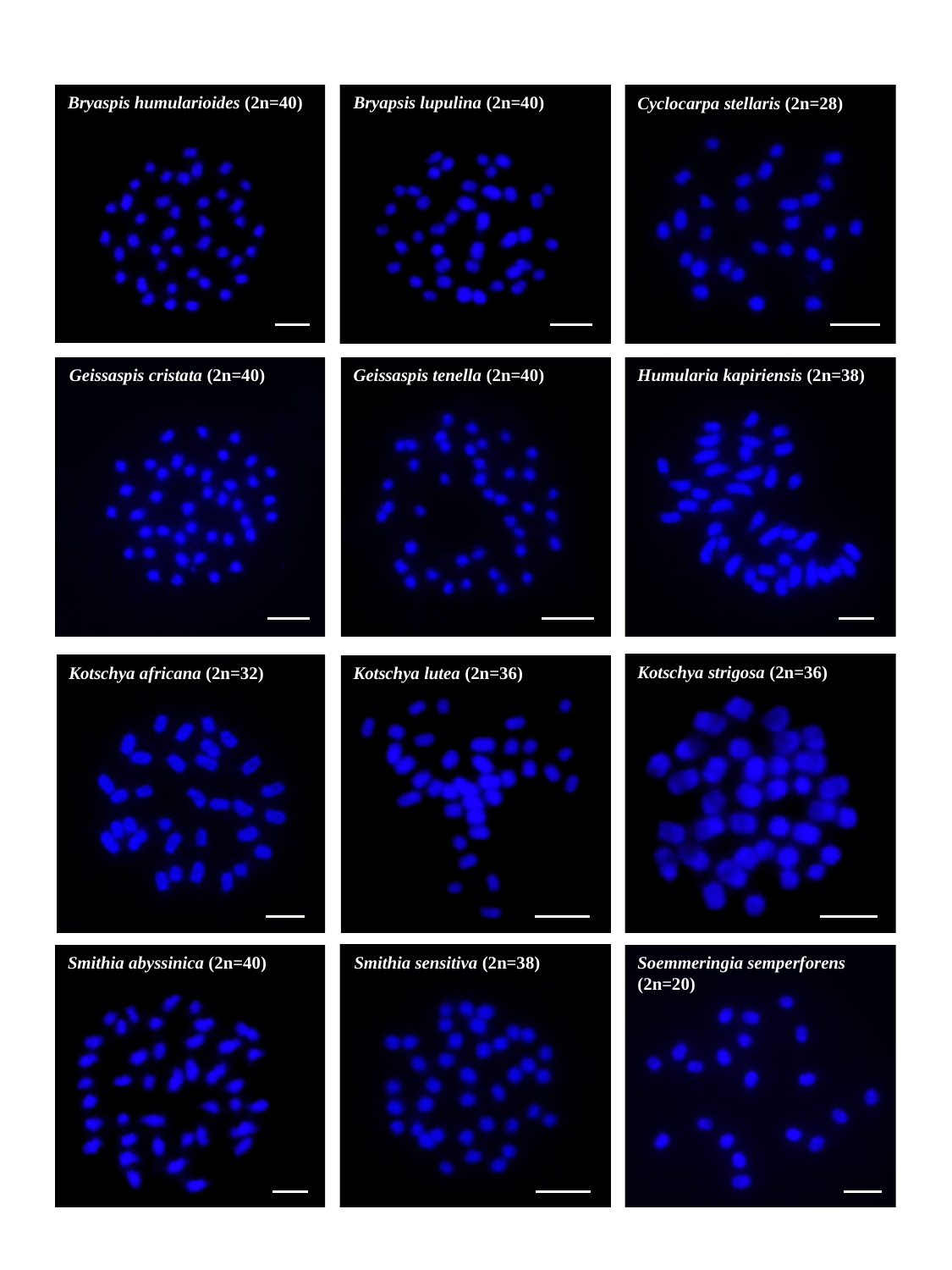

Bryaspis humularioides (2n=40)
Bryapsis lupulina (2n=40)
Cyclocarpa stellaris (2n=28)
Geissaspis tenella (2n=40)
Geissaspis cristata (2n=40)
(Gtenella)
Humularia kapiriensis (2n=38)
Kotschya strigosa (2n=36)
Kotschya africana (2n=32)
Kotschya lutea (2n=36)
Smithia abyssinica (2n=40)
Smithia sensitiva (2n=38)
Soemmeringia semperforens (2n=20)
