# Supplementary material for: A phylogenetic framework of the legume genus Aeschynomene for comparative genetic analysis of the Nod-dependent and Nod-independent symbioses
Source: BMC Plant Biol. 2018 Dec 5;18:333. doi: 10.1186/s12870-018-1567-z (PMC6282307; doi:10.1186/s12870-018-1567-z)
Supplement: Supplementary file 11 — Figure S8. Ancestral state reconstruction of adventive root primordia in the genus Aeschynomene and allied genera. Ancestral state reconstruction was estimated in SIMMAP software using the 50% majority-rule topology obtained by Bayesian analysis of the combined ITS + matK sequences. Data on the adventitious root primordia come from the present analysis and pertinent previously published data. Presence or not of adventitious root primordia is indicated by different colors. (PPTX 96 kb) [file 12870_2018_1567_MOESM11_ESM.pptx]

## Slide 1
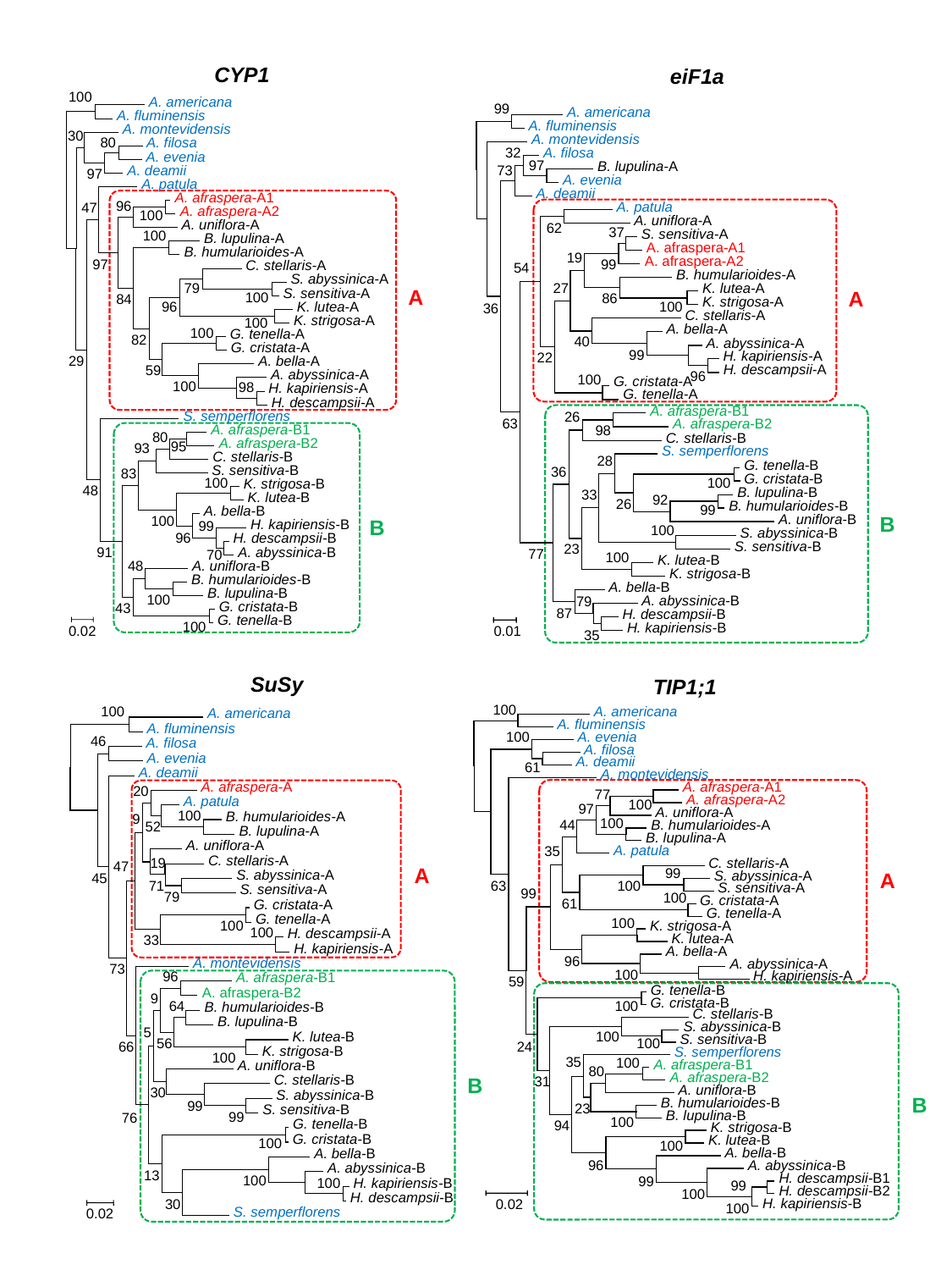

CYP1
eiF1a
100
 A. americana
 A. fluminensis
 A. montevidensis
30
80
 A. filosa
 A. evenia
 A. deamii
97
 A. patula
 A. afraspera-A1
96
47
 A. afraspera-A2
100
 A. uniflora-A
100
 B. lupulina-A
 B. humularioides-A
97
 C. stellaris-A
 S. abyssinica-A
79
 S. sensitiva-A
100
84
 K. lutea-A
96
 K. strigosa-A
100
100
 G. tenella-A
82
 G. cristata-A
29
 A. bella-A
59
 A. abyssinica-A
100
98
 H. kapiriensis-A
 H. descampsii-A
 S. semperflorens
 A. afraspera-B1
80
 A. afraspera-B2
95
93
 C. stellaris-B
 S. sensitiva-B
83
100
 K. strigosa-B
48
 K. lutea-B
 A. bella-B
100
 H. kapiriensis-B
99
96
 H. descampsii-B
 A. abyssinica-B
91
70
 A. uniflora-B
48
 B. humularioides-B
 B. lupulina-B
100
 G. cristata-B
43
 G. tenella-B
100
0.02
A
B
99
 A. americana
 A. fluminensis
 A. montevidensis
 A. filosa
32
97
 B. lupulina-A
73
 A. evenia
 A. deamii
 A. patula
 A. uniflora-A
62
37
 S. sensitiva-A
 A. afraspera-A1
19
 A. afraspera-A2
99
54
 B. humularioides-A
27
 K. lutea-A
A
86
 K. strigosa-A
100
36
 C. stellaris-A
 A. bella-A
40
 A. abyssinica-A
99
 H. kapiriensis-A
22
 H. descampsii-A
96
100
 G. cristata-A
 G. tenella-A
 A. afraspera-B1
26
63
 A. afraspera-B2
98
 C. stellaris-B
 S. semperflorens
28
 G. tenella-B
36
 G. cristata-B
100
 B. lupulina-B
33
92
26
 B. humularioides-B
99
B
 A. uniflora-B
100
 S. abyssinica-B
 S. sensitiva-B
23
77
100
 K. lutea-B
 K. strigosa-B
 A. bella-B
 A. abyssinica-B
79
87
 H. descampsii-B
 H. kapiriensis-B
0.01
35
SuSy
TIP1;1
100
 A. americana
100
 A. americana
 A. fluminensis
46
 A. filosa
 A. evenia
 A. deamii
 A. afraspera-A
20
 A. patula
100
 B. humularioides-A
9
52
 B. lupulina-A
 A. uniflora-A
 C. stellaris-A
19
A
47
 S. abyssinica-A
45
71
 S. sensitiva-A
79
 G. cristata-A
 G. tenella-A
100
100
 H. descampsii-A
33
 H. kapiriensis-A
 A. montevidensis
73
96
 A. afraspera-B1
 A. afraspera-B2
9
64
 B. humularioides-B
 B. lupulina-B
5
 K. lutea-B
56
66
 K. strigosa-B
100
 A. uniflora-B
B
 C. stellaris-B
30
 S. abyssinica-B
99
 S. sensitiva-B
99
76
 G. tenella-B
 G. cristata-B
100
 A. bella-B
 A. abyssinica-B
13
100
 H. kapiriensis-B
100
 H. descampsii-B
30
 S. semperflorens
0.02
 A. fluminensis
 A. evenia
100
 A. filosa
 A. deamii
61
 A. montevidensis
 A. afraspera-A1
77
 A. afraspera-A2
100
97
 A. uniflora-A
100
44
 B. humularioides-A
 B. lupulina-A
 A. patula
35
 C. stellaris-A
A
99
 S. abyssinica-A
63
100
 S. sensitiva-A
99
100
 G. cristata-A
61
 G. tenella-A
100
 K. strigosa-A
 K. lutea-A
 A. bella-A
96
 A. abyssinica-A
100
 H. kapiriensis-A
59
 G. tenella-B
 G. cristata-B
100
 C. stellaris-B
 S. abyssinica-B
100
 S. sensitiva-B
100
24
 S. semperflorens
35
100
 A. afraspera-B1
80
 A. afraspera-B2
31
 A. uniflora-B
B
 B. humularioides-B
23
 B. lupulina-B
100
94
 K. strigosa-B
 K. lutea-B
100
 A. bella-B
96
 A. abyssinica-B
 H. descampsii-B1
99
99
 H. descampsii-B2
100
 H. kapiriensis-B
0.02
100
